# Thousands of Rab GTPases for the Cell Biologist

**DOI:** 10.1371/journal.pcbi.1002217

**Published:** 2011-10-13

**Authors:** Yoan Diekmann, Elsa Seixas, Marc Gouw, Filipe Tavares-Cadete, Miguel C. Seabra, José B. Pereira-Leal

**Affiliations:** 1 Instituto Gulbenkian de Ciência, Oeiras, Portugal; 2 Centro de Estudos de Doenças Crónicas (CEDOC), Faculdade de Ciências Médicas da Universidade Nova de Lisboa, Lisboa, Portugal; Stockholm University, Sweden

## Abstract

Rab proteins are small GTPases that act as essential regulators of vesicular trafficking. 44 subfamilies are known in humans, performing specific sets of functions at distinct subcellular localisations and tissues. Rab function is conserved even amongst distant orthologs. Hence, the annotation of Rabs yields functional predictions about the cell biology of trafficking. So far, annotating Rabs has been a laborious manual task not feasible for current and future genomic output of deep sequencing technologies. We developed, validated and benchmarked the Rabifier, an automated bioinformatic pipeline for the identification and classification of Rabs, which achieves up to 90% classification accuracy. We cataloged roughly 8.000 Rabs from 247 genomes covering the entire eukaryotic tree. The full Rab database and a web tool implementing the pipeline are publicly available at www.RabDB.org. For the first time, we describe and analyse the evolution of Rabs in a dataset covering the whole eukaryotic phylogeny. We found a highly dynamic family undergoing frequent taxon-specific expansions and losses. We dated the origin of human subfamilies using phylogenetic profiling, which enlarged the Rab repertoire of the Last Eukaryotic Common Ancestor with Rab14, 32 and RabL4. Furthermore, a detailed analysis of the Choanoflagellate *Monosiga brevicollis* Rab family pinpointed the changes that accompanied the emergence of Metazoan multicellularity, mainly an important expansion and specialisation of the secretory pathway. Lastly, we experimentally establish tissue specificity in expression of mouse Rabs and show that neo-functionalisation best explains the emergence of new human Rab subfamilies. With the Rabifier and RabDB, we provide tools that easily allows non-bioinformaticians to integrate thousands of Rabs in their analyses. RabDB is designed to enable the cell biology community to keep pace with the increasing number of fully-sequenced genomes and change the scale at which we perform comparative analysis in cell biology.

## Author Summary

Intracellular compartmentalisation via membrane-delimited organelles is a fundamental feature of the eukaryotic cell. Understanding its origins and specialisation into functionally distinct compartments is a major challenge in evolutionary cell biology. We focus on the Rab enzymes, critical organisers of the trafficking pathways that link the endomembrane system. Rabs form a large family of evolutionarily related proteins, regulating distinct steps in vesicle transport. They mark pathways and organelles due to their specific subcellular and tissue localisation. We propose a solution to the problem of identifying and annotating Rabs in hundreds of sequenced genomes. We developed an accurate bioinformatics pipeline that is able to take into account pre-existing and often inconsistent, manual annotations. We made it available to the community in form of a web tool, as well as a database containing thousands of Rabs assigned to sub-families, which yields clear functional predictions. Thousands of Rabs allow for a new level of analysis. We illustrate this by characterising for the first time the global evolutionary dynamics of the Rab family. We dated the emergence of subfamilies and suggest that the Rab family expands by duplicates acquiring new functions.

## Introduction

Intracellular compartmentalisation is found in all cellular lifeforms, yet eukaryotes have evolved extensive membranous compartments unique to this domain of life. Protein trafficking pathways accomplish the movement of cellular components like proteins and lipids between the cellular compartments. These essential pathways play house-keeping roles, such as transport of proteins destined for secretion to the plasma membrane via the secretory pathway, or recycling of membrane receptors via the endocytic pathway. In addition, they play a variety of specialised roles, such as bone resorption in osteoclasts, pigmentation in melanocytes and antigen presentation in immune cells. Malfunction of protein trafficking components leads to a large number of human diseases, ranging from hemorrhagic disorders and immunodeficiencies to mental retardation and blindness [1]–[4], as well as cancer [5]–[9]. Furthermore, protein trafficking pathways are frequently exploited by human pathogens to gain entry and survive within host cells [10]–[13].

The endomembrane system accounts for a large fraction of the protein coding sequences in eukaryotic genomes [14], and a plethora of data on molecules and interactions in different model organisms is available. However, it is unclear how these data map across organisms, and how general the mechanisms characterised in single species are. To answer these question we need to understand the evolution of the protein trafficking pathways and organelles. An evolutionary framework for protein trafficking is particularly important given the overwhelming accumulation of genomes, many from pathogenic organisms. Their comparative analysis can distinguish conserved from taxon-specific machineries, with clear practical applications. For example, conservation of genes led to the discovery of novel components and mechanisms in ciliogenesis [15], whereas the presence of taxon-specific pathways allowed the identification of Fosmidomycin as a potential antimalarial drug [16]. Studying the evolution of protein trafficking is essential to understand the origins of eukaryotes. Comparative genomics and phylogenetics have established that the Last Eukaryotic Common Ancestor (LECA) already had a complex membrane trafficking system [17] including most types of extant molecular components [18]. These are believed to have expanded by duplication and specialisation giving rise to the full diversity of organelles and trafficking pathways observed today (see [17] for a detailed description of this evolutionary scenario).

Rabs are central regulators of protein trafficking. They are small GTPases that work as molecular switches to regulate vesicle budding, motility, tethering and fusion steps in vesicular transport [19]. Most recently the authors of [20] also linked Rabs to membrane fission. They recruit molecular motors to organelles and transport-vesicles, coordinate intracellular signalling with membrane trafficking, organise distinct sub-domains within membranous organelles and play a critical role in the definition of organelle identity (recently reviewed in [21]). Rab subfamilies localise to distinct cellular locations, and regulate trafficking in a pathway-, organelle- and tissue-specific manner. This makes them ideal markers for the majority of trafficking-processes and compartments. Among trafficking-associated proteins, the Rab family expanded most in evolution [17], [22], suggesting that it provided the primary diversification element in the evolution of trafficking [22]. An important feature of the Rab family is that Rab orthologs tend to perform similar functions even in divergent taxa. For example, the mouse Rab1 has been shown to be able to functionally replace its ortholog YPT1 in yeast [23]. Hence assigning a Rab to a known and functionally described subfamily, *e.g.* Rab1, is a strong functional prediction, *i.e.* functioning in the early secretory pathway in the case of Rab1. Together with the ability to classify them into subfamilies based on sequence alone, this allows to establish the presence or loss of pathways and organelles solely based on the annotation of the Rab repertoire—a procedure we subsequently refer to as Rab profiling.

Previously, we defined criteria to identify and classify Rab proteins [24], which have been used as a basis for detailed manual analysis of the Rab families in a variety of organisms [25]–[33]. However, manual identification of Rab repertoires is tedious and time-consuming and not compatible with the deluge of fully sequenced eukaryotic genomes that new sequencing technologies are generating. We thus need to develop methods that enable the automated annotation of Rab proteins. Several characteristics of the Rab family make this a challenging bioinformatics problem. First, there is a strong non-specific signal from GTPase motifs spread throughout the protein sequence [34], which makes it hard to distinguish Rabs from other small GTPases. Second, the Rab family is large due to extensive duplication in several branches of the eukaryotic tree (*e.g.* [28], [29]). Together with high sequence similarity amongst Rabs this causes difficulties to correctly classify Rabs into subfamilies and to further discern yet unseen subfamilies. Lastly, any automated scheme has to respect and perpetuate as much as possible the current naming conventions, despite any inconsistencies stemming from the decentralised nature of scientific discovery and the huge bias of existing annotations towards Ophistokonts. This requires a flexible, learning scheme both able to cope with the contingency of the field and to easily incorporate new naming consensuses.

Here, we overcame these problems and developed an automated bioinformatic pipeline for the identification and classification of Rabs. We termed our pipeline the ‘Rabifier’, which we describe, validate and benchmark. Using our tool, we cataloged nearly 8.000 Rabs from 247 genomes covering the major taxa of the eukaryotic tree, which we make available along with our pipeline at www.RabDB.org.

Based on this comprehensive dataset of Rab proteins, we describe and analyse the evolution of Rabs. We found a highly dynamic family undergoing frequent taxon-specific expansions and losses. We extend the Rab repertoire previously reported to have been present in the LECA, identify the changes in the Rab family that accompanied the emergence of multicellularity and show that neo-functionalisation best explains the emergence of new human Rab subfamilies.

## Results/Discussion

### The Rabifier

**Figure 1:**
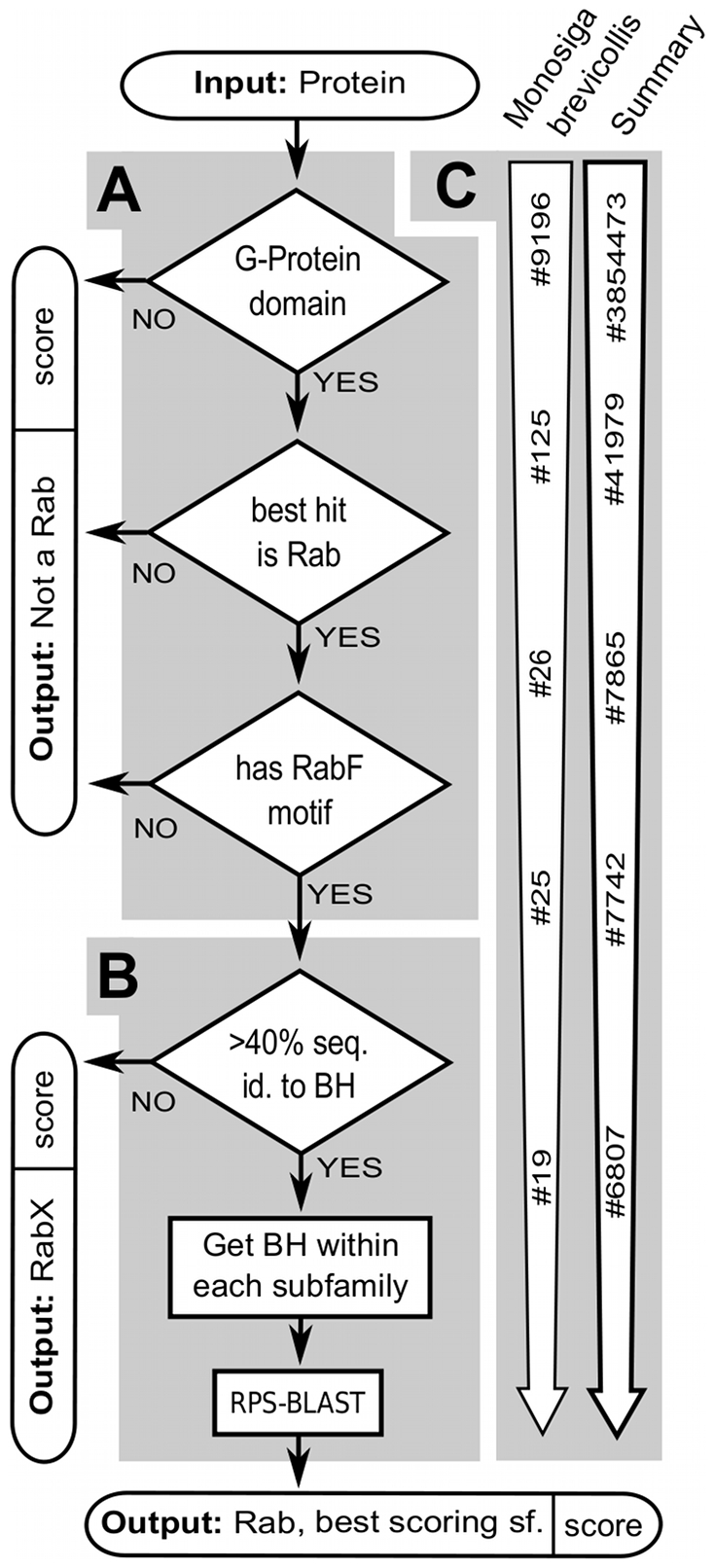
Flowchart of the Rabifier. (**A**) Identification- and (**B**) classification-procedure implemented by the Rabifier, see **Results and Discussion** for details on the two phases. Panel (**C**) shows descriptive statistics from the application of the Rabifier to 247 genomes in the Superfamily database, and details about *Monosiga brevicollis*. Abbreviations: best BLAST hit (BH) [37], Rab family motif (RabF) [24], reverse Ψ-BLAST (RPS-BLAST) [38], subfamily (sf.), Rab not classified to any subfamily within our internal reference set (RabX).

We implemented a bioinformatics pipeline to identify and classify Rab GTPases in any set of protein sequences independently of taxonomical information, which we term ‘Rabifier’. The Rabifier proceeds in two major phases, which are schematised in **Figure 1**. First, it decides whether a protein sequence belongs to the Rab family, *i.e.* that it is not a Ras, a Rho, etc., and in the second phase it classifies the predicted Rab sequence into a Rab subfamily (*e.g.* Rab1). We describe the rationale for this procedure below—technical details are given in **Materials and Methods** and **Text S1**.

Phase 1 (**Figure 1A**), which classifies protein sequences to the Rab family, proceeds in three stages. First, we check that the protein has a G-protein family domain. As the presence of such a domain can be decided with near certainty, this step drastically reduces the number of candidate Rabs while not excluding any real Rab. In order to do so, we align the sequence against a profile Hidden Markov Model (HMMs) [35] describing the known GTPase structures, as provided by the Superfamily database [36]. Secondly, we search for local sequence similarity by performing a BLASTp [37] query against an internal reference set of manually curated GTPases and discard the protein if it is most similar to a GTPase other than a Rab. At this stage of the workflow, the majority of non-Rab sequences has already been rejected (see **Figure 1C**, where the number of sequences that transition between these phases is shown for *M. brevicollis* and for a database of 247 genomes described below). However, small GTPases are so similar to each other that a residual amount of false positives still remains undetected. We remove them in the third stage, where we scan the sequence for the presence of at least one of five characteristic RabF motifs defined in [24]. If no motif is found, it is concluded that the protein cannot be a Rab and rejected. Remaining sequences are all assigned to the Rab family at an individual confidence level computed for each Rab. The confidence score is derived from the combination of the individual statistics generated by the three stages according to a procedure described in **Text S1**.

The second phase (**Figure 1B**) proposes a classification into one of the Rab subfamilies present in our internal reference set, or suggests no similarity to any of those. It proceeds in two stages. First, we test whether the Rab respects a 40% identity cut-off to its BH that prevents assignment of too disparate sequences to any of the pre-defined subfamilies. If the cut-off is met, a classification is proposed, if not, the Rab is classified as belonging to the undetermined subfamily RabX. The use of a 40% threshold is supported in **Figure S1**, and has previously been employed for example in [30]. The actual subfamily classification is based on the computation of a likelihood score for each of the subfamilies in our reference set. Intuitively, the protein is classified as belonging to the highest scoring subfamily, however, all scores are kept and thus provide an estimate of the relative uncertainty associated with each call. Like the Rab family score generated in the first phase of the Rabifier, the computation integrates output statistics from different tools, namely from local alignments via BLAST and from alignments using reverse Ψ-BLAST (RPS-BLAST [38]). Similar to HMMs, RPS-BLAST compares a sequence against a summary of a set of sequences, in our case summaries of all sequences in our reference set belonging to a single Rab subfamily, and measures how likely the input belongs to any the subfamilies. This way we take information from all sequences in the internal reference set into account. For details on the procedure check **Materials and Methods** and Supplementary Methods **Text S1**.

### Validation of the Rabifier classifications and design

Any new methodology has to be validated. Ideally this is based on a test data set fulfilling three requirements: the test data is correctly and comprehensively annotated with those features the tool automatically detects, it is large enough to provide robust statistics, and it covers the entire range of possible inputs the tool might encounter in its real-world application, at best even respecting the expected proportions of worst- to best-case inputs. In our case, no dataset is available which fulfils the three requirements simultaneously: Rab repertoires are only available for a limited number of organisms which are not evenly distributed across eukaryotic phylogeny, and whose annotation was manually performed by different groups, hence may be inconsistent or even incorrect (in some cases a ‘correct’, *i.e.* consensual, classification might not even exist).

**Figure 2:**
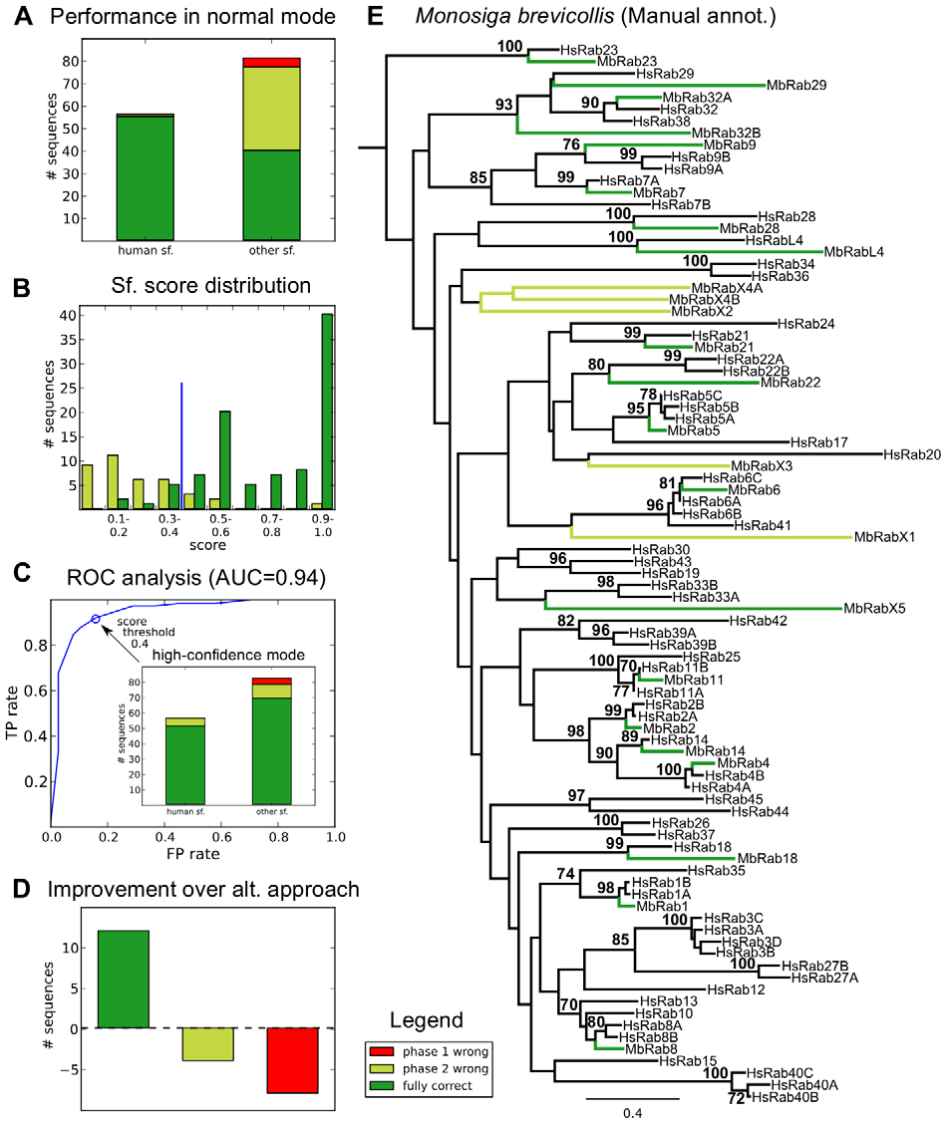
Validation and benchmarking of the Rabifier. (**A**) summarises the validation in normal mode, *i.e.* without taking the subfamily score produced by Rabifier into account, against the Rab families of *Trypanosoma brucei* [32], *Entamoeba histolytica* [30] and *Monosiga brevicollis*, which we annotated in (**E**). Three quantities needed to judge the performance of the Rabifier are shown for Rabs belonging to human and other subfamilies separately: sequences erroneously classified as not being a Rab by the Rabifier (red), sequences correctly identified as Rabs, however, wrongly classified at subfamily level (light green), and those which were entirely correct (dark green). (**B**) displays the distribution of confidence scores associated to each subfamily call, respecting the same colour code as above. The blue line indicates the threshold which we propose on default, and below which subfamily classification may be rejected and treated as a undefined RabX. That choice is based on the ROC-curve [113] analysis shown in (**C**), which plots the true positive rate against the false positive rate for each possible confidence threshold [113] and provides a combined measure of the accuracy of a classifier (Area under the curve, AUC [39]). The effect of choosing an 0.4 confidence threshold (blue circle) on the classification accuracy, *i.e.* running the Rabifier in high confidence mode, is shown in the inlay. (**D**) plots the improvement in terms of the three quantities discussed above the Rabifier achieves compared to an alternative strategy (see **Results and Discussion** for details on its implementation). (**E**) Phylogenetic tree of the human and *M. brevicollis* Rab family on which the manual classification of the latter Rab family was based (bootstrap support above 70% shown). Colours indicate the results of the corresponding automated annotation for that specific sequence. Abbreviations: subfamily (sf.), annotation (annot.).

In the absence of a suitable validation dataset, we opted to validate the Rabifier against the manually curated Rab families of three organisms representing distinct worst case scenarios for the Rabifier (**Figure 2A–C**, see **Table S1** for a list of all sequences used). This ensures that the validation is meaningful, as it provides a strict lower bound on the expected performance in everyday use. First, we chose the Excavate *Trypanosoma brucei* [32], which is one of the most distantly related organism to our reference sequences, which are dominated by Ophistokonts (an unranked scientific classification sometimes also called ‘Fungi/Metazoa group’). The second is *Entamoeba histolytica* [30], a Unikont from the phylum of Amoebozoa that is thus marginally closer to the sequences that dominate our reference database, but has a heavily expanded and diverse Rab repertoire which makes it challenging to assign Rab subfamilies. The third organism, *Monosiga brevicollis* from the class of Choanoflagellates, was chosen as a representative of a phylum (Choanozoa) for which no information on the Rab family is available yet. In this third case, we compare the automated predictions against a manual analysis we performed in this study (**Figure 2E**), and which we will discuss below.

The first aspect we assessed is the ability of the Rabifier to distinguish Rabs from other GTPases (summarised in **Figure 2A**). We present the Rabifier with the set of GTPases from the above organisms and count how often we miss a Rab (false negative—FN), and how often we incorrectly classify a non-Rab as a Rab (false positive—FP). For *T. brucei*, we correctly classified 101 out of 102 GTPases as being a Rab or not, 292 out of 295 in *E. histolytica* and finally all 125 GTPases in *M. brevicollis*. Altogether, we have no FP and 4 FN, which means that for this particular set of genomes we make correct decisions about whether a protein is a Rab in 99.2% of the cases with no differences amongst the organisms. In order to understand the sources of the misannotations at family level, we inspected the false negatives individually. The Rabifier disagrees with the manual curation of [32] in *T. brucei* for TbRabX3, a RabL2-like protein, that is counted as a false negative. We explicitly added RabL2 sequences to our negative data set as we do not consider these proteins as members of the Rab family (see **Materials and Methods**). The remaining disagreements between the Rabifier and the manual annotations are three false negative proteins in *E. histolytica* in which we cannot find any detectable RabF motif, and one protein which has no similarity to any member of our reference dataset of small GTPases. We conclude that these proteins are likely misclassified in [30], and hence that the above failures of the Rabifier to identify Rabs are artificially introduced by our validation procedure.

Secondly, we established the accuracy by which a given Rab sequence is assigned to the right subfamily (summarised in **Figure 2A**). Concretely, for those sequences which were correctly identified as Rabs, we checked whether the proposed subfamily agreed either with the public annotation or our own one for *M. brevicollis*. We distinguished between two operating modes of the Rabifier: a normal one which does not consider the confidence levels the Rabifier attributes to its classifications, and a high-confidence mode which accepts only the high-confidence annotations above a certain confidence threshold, whereas those below are classified as belonging to the undetermined subfamily RabX. Ignoring the information provided by the classification confidence, we correctly called 16 out of 17 Rabs for *T. brucei*, 59 out of 91 in *E. histolytica* and 20 out of 25 for *M. brevicollis*, leading to an overall fraction of 71.4% correct decisions (79.7% on average per organism). However, if one defines a threshold below which a classification is systematically considered as belonging to the undefined subfamily RabX, the accuracy can be substantially improved. To illustrate this, **Figure 2B** displays the distribution of scores associated to correct and wrong calls, which shows that wrong calls clearly have lower confidence scores on average. In order to test for all possible thresholds exploiting this difference, we performed a ROC curve analysis presented in **Figure 2C**. This machine learning technique allows to summarise and quantify the classification performance for all thresholds (Area Under the Curve (AUC) [39], here 0.94), and enables to objectively choose a threshold providing an optimal TP/FP-tradeoff. Here, we opted for 0.4, which we propose as a default choice for the interpretation of the Rabifier's results. Yet, the use of this threshold is not fixed as it may vary depending on the dataset, and can be freely modified by users of the Rabifier. The consequences of applying a cutoff on the classification accuracy are quantified by the inlay in **Figure 2C**: only trusting calls with confidence higher or equal to 0.4 greatly reduces the amount of misclassified Rabs from non-human subfamilies and improves the overall accuracy to 90% (92.01% on average per organism).

In summary, we conclude that our workflow is able to correctly discern Rabs from other GTPases. Furthermore, calls both at family and subfamily level have an associated confidence score which correctly captures uncertainty in the decision. Relying on the information provided by the confidence level, the Rabifier suggests correct subfamilies around 90% of the time even in difficult and phylogenetically isolated cases.

### Benchmarking the Rabifier

After having established the correctness of our procedure, we wished to assess the improvement it represents over possible alternative large-scale approaches in an objective manner. This excludes benchmarking against methods for example based on phylogenetic trees, as reasoning over them is difficult to automate and not feasible for thousands of sequences.

We chose to compare the Rabifier to the Conserved Domain Database at the NCBI [40], the only resource we are aware of that specifically scores for RabF motifs. To this end, we implemented an alternative decision scheme which given a protein retrieves the protein name and CDD domain annotation of its BH in the NCBI protein database. Note that if the protein is in the NCBI database, the BH retrieves the protein itself. As for the choice of genome, the Rabifier has to be benchmarked against an organism whose Rab family has not been manually curated, as our alternative procedure would simply retrieve that annotation. Moreover, an organism from a taxon which is both close to Metazoa and for which no information on the Rab family exists best ensures an unbiased measurement. These requirements are met by the Choanoflagellate *M. brevicollis*, which we analysed ourselves and is thus an ideal candidate for a direct comparison.

The results of this experiment are detailed in **Figure 2D** (see also **Table S1**). As above, we distinguished between the ability to discern Rabs from other GTPases and to actually propose the correct subfamily for a given Rab. First, while the Rabifier achieved 100% accuracy in separating Rabs from other GTPases in *M. brevicollis*, the alternative strategy—although not introducing false positives—misses 8 of 25 Rabs leading to an overall drop in sensitivity. On top of these 8 sequences, the Rabifier correctly suggests subfamilies for 4 further proteins wrongly classified by the alternative strategy, leading to an overall difference of 12 sequences correctly classified only by the Rabifier.

Thus, our annotation pipeline represents a significant improvement over currently available large scale approaches, both in terms of sensitive identification of Rabs and especially with regards to the difficult automatic classification of Rabs into subfamilies.

### Availability of the Rabifier and its predictions

**Figure 3:**
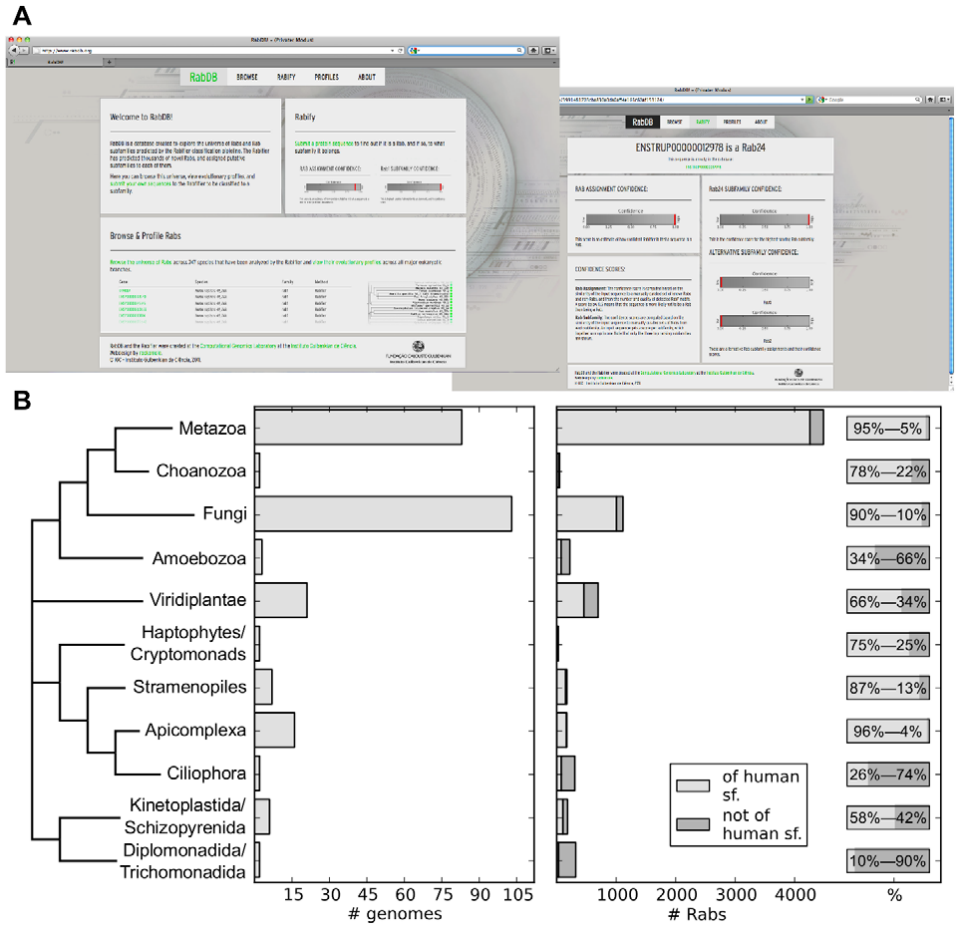
Resources we make available. (**A**) Snapshots of the database www.RabDB.org which provides public access to the results of the Rabifier applied to the Superfamily database [41] and the online version of the Rabifier. (**B**) Statistics of the current content of www.RabDB.org in terms of number of genomes (left), absolute number of Rabs either belonging to a subfamily also present in humans or not (middle), and the relative fraction of the two types of Rabs for a given branch (right). The cladogram (*i.e.* the branch length are arbitrary, see [114]) of the eukaryotic taxa is derived from [115].

In order to make our pipeline useful to the cell biology community interested in Rabs, we provide access to the Rabifier in form of a web tool (**Figure 3A**). Via the graphical interface users can submit up to five protein sequences at a time, and the classifications generated by our workflow are returned together with their associated degree of confidence. We envisage users who want to quickly generate hypotheses about one or a few candidate proteins. Users wishing to classify more sequences are encouraged to contact us. We emphasise that the Rabifier works without need for phylogenetic information about the input, hence any set of protein sequences can be submitted.

In addition, we generated a database of nearly 8,000 classified Rab sequences in 247 eukaryotic genomes, which we make publicly available at www.RabDB.org (**Figure 3A**) together with basic browsing and visualisation tools. Our database is built on top of the Superfamily database [41] (September 2009 release), which allows us to follow its release cycle and include predictions for all newly sequenced genomes contained therein. **Figure 3B** details the phylogenetic distribution of genomes in RabDB and the number of Rabs we predict in each of those eukaryotic branches. The correctness of the content in www.RabDB.org is not manually confirmed systematically. However, we constantly inspect and manually curate the generated predictions and update our internal reference database accordingly. Furthermore, we provide users the possibility to notify us of a potential mis-annotation found in the database such that we can correct the classification of the Rab in question. These measures further enhance the expected quality of future releases of www.RabDB.org.

### New hypothetical subfamilies

**Figure 4:**
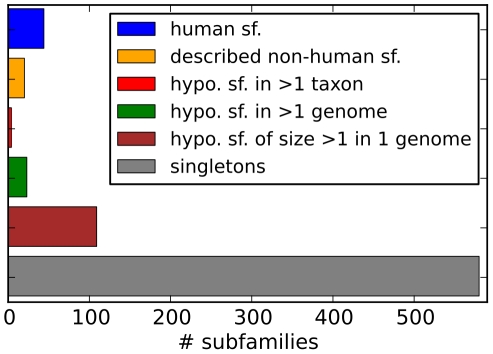
Rab subfamilies in or dataset. Number of different Rab subfamilies found in our dataset. Human sf. are shown in blue, and other known sf. in orange. The last four categories are hypothetical subfamilies we propose in the context of this paper (see **Materials and Methods** for details on the procedure): subfamilies whose members span more than one taxon (red), those spanning more than on genome (green), subfamilies with several members yet only present in one organism (brown) and finally singletons (grey) which are not similar to any other known Rab. All members and subfamilies can be browsed in our website at www.RabDB.org. Abbreviations: hypothetical (hypo.), subfamily (sf.).

As can be noticed from **Figure 3B**, the Rabifier detected a large number of Rabs not belonging to any subfamily represented in our reference set, *i.e.* most subfamilies which have been described before. By definition these sequences show no similarity to any functionally characterised Rab, hence a bioinformatic annotation is not possible. However, in order to structure the space of new sequences and provide a starting point to study this yet unexplored diversity, we clustered these Rabs with respect to their sequence identity and propose several hypothetical Rab subfamilies (see **Material and Methods** for details). The result of this procedure is shown in **Figure 4**, which details the amount of hypothetical subfamilies according to the breadth of their occurrence (see **Figure S7** for an overview of the amount of Rabs falling into each of these classes). We integrated these new subfamilies both in our database, where they can be browsed with help of the visualisation tools we provide, and in the online version of the Rabifier. Note that in addition to these new hypothetical subfamilies we still find hundreds of Rabs that we cannot group with others. Those may result from erroneous gene models in less well curated genomes, represent cases where our simple clustering procedure failed, or indeed be *bona fide* singletons. A detailed phylogenetic analysis may be required to resolve these cases which is out of the scope of this study.

### Global dynamics of the Rab sequence space

**Figure 5:**
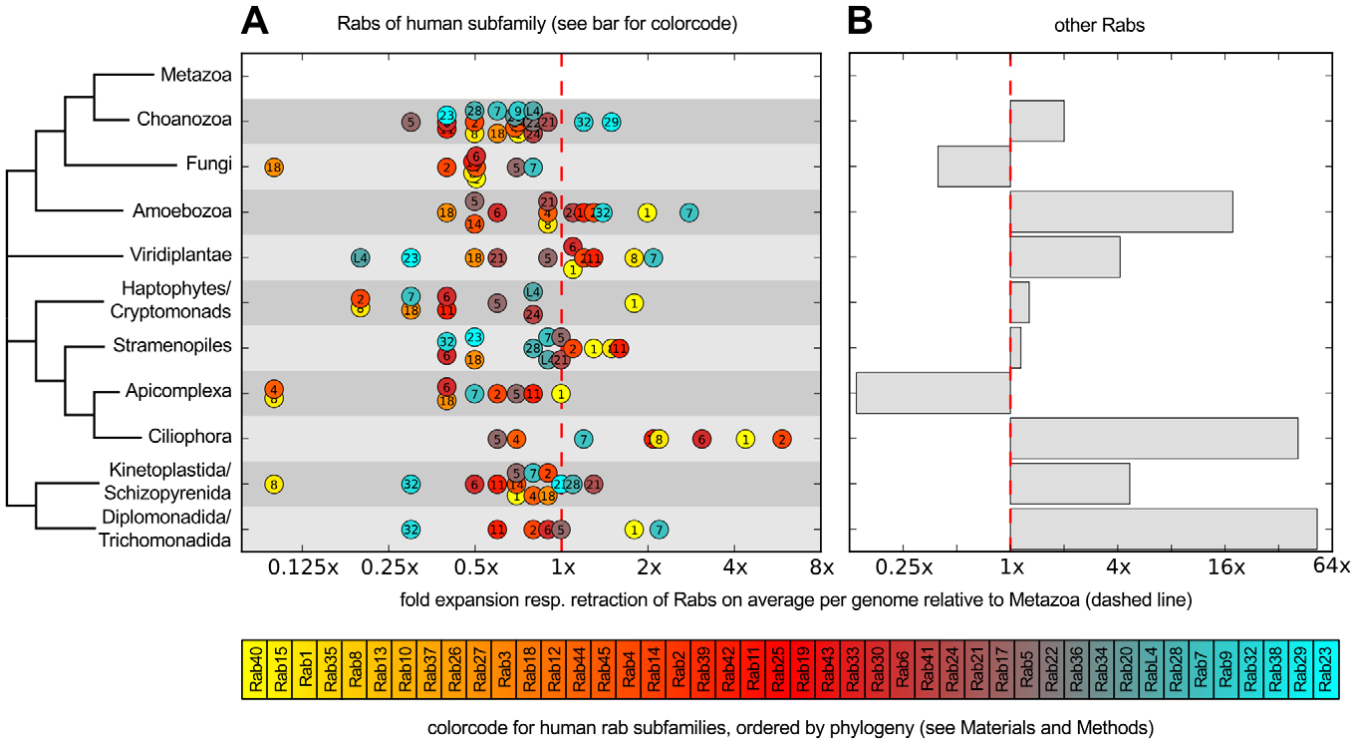
Rab subfamily expansions relative to Metazoa in a dataset of 247 genomes. For each of the eukaryotic taxa (as derived from [115]), (**A**) displays the relative size compared to Metazoa of each human Rab subfamily on average per genome. The dashed line represents the average in Metazoan genomes, *i.e.* any circle lying on that line represents a human subfamily that has the same amount of members on average per genome than on average in Metazoa. Similarly, any circle to the left represents a subfamily that is smaller compared to Metazoa, finally, all on the right are expanded compared to the Metazoan average. Note that the axis are in logarithmic scale. In addition to the numbers indicating the human Rab subfamily, a colour code to distinguish subfamilies is shown below, where similar colours indicate proximity in the phylogenetic tree of human Rabs. The same plot for all other Rabs is shown in (B), again on a logarithmic scale. All sequences used are accessible at www.RabDB.org. Abbreviations: subfamily (sf.).

A dataset of 8,000 Rabs allows us to take a global view of the Rab sequence space, and to address previously inaccessible questions. Here, we investigate the patterns of Rab repertoire expansion in the eukaryotic tree (**Figure 5**). Expansion of certain protein families has been found to correlate with organismal complexity [42]. The anecdotal evidence of Rab profiles in different organisms suggests at least three possible scenarios: a conserved core of Rabs present in all organisms; tinkering with a core of subfamilies by taxon- or species-specific expansions of existing subfamilies; a major variation of the Rab machinery with taxon- or species-specific Rab repertoires. We asked whether any such scenario is apparent for the Rab family across the eukaryotic tree, or if different ones predominate in different branches.

We observe a tremendous heterogeneity in the sizes of Rab repertoires, ranging from 5 to several hundreds of Rabs in *Encephalitozoon cuniculi* and *Trichomonas vaginalis* respectively. Genomic analyses have shown a general trend for more and larger families in bigger genomes [43], [44]. In the case of Rabs, linear regression over all taxa reveals that genome size explains roughly 60% of the observed variance in numbers of Rabs in an organism (**Figure S2**). However, due to the current bias in fully sequenced genomes towards Ophistokonts (compare **Figure 3B**), it is unclear whether these numbers will remain as such in the future. We find that closely related organisms tend to have similar Rab repertoires in size, but at the level of phyla we encounter marked differences indicating taxon-specific adaptations. For example, although Ciliophora and Apicomplexa belong to the same superphylum (Alveolata), these sister phyla show very different repertoires, highly expanded in the first case, and streamlined in the second. The smaller Rab repertoires in Apicomplexan genomes, mostly dominated by intracellular parasites, may be due to secondary gene loss, similar to that reported in bacterial intracellular parasites and endosymbionts [45] and in the obligate intracellular parasitic Microsporidia [45]. Another example of reduction of Rab repertoires is observed in the fungal branch, as we reported previously [26] and now confirm based on an extended set of 103 genomes. It is noteworthy that Fungi are Unikonts, a taxon which comprises Metazoa and Amoebozoa, *i.e.* branches that appeared to have suffered independent expansions of their Rab repertoires [24], [30]. We observe large expansions in Diplomonadida/Trichomonadida, Ciliophora and Amoebozoa. Much of these expansions are accounted for by species-specific subfamilies (see **Figure 4**). This demonstrates that there is frequent invention of new Rabs, perhaps in a taxon-specific manner—a hypothesis that will have to await broader sampling of the genomes space to be tested in most taxa. On the other hand, inspection of **Figure 5** reveals that for those Rabs that can be classified, different subfamilies expanded in each branch of the tree. For example, Rab7 forms the largest subfamily in Diplomonadida/Trichomonadida and Amoebozoa, whereas Ciliophora's most expanded subfamily is Rab2. This suggests that these are independent expansions, which has already been observed for example within the Rab5 subfamily [26], [46]. Note that we repeated these analyses for different confidence cutoffs and observed no significant consequences on the broad picture.

In summary, the global evolution of Rab repertoires is highly dynamic with frequent taxon-specific subfamily expansions, gain of new Rabs and losses. Hence, we observe a scenario where a core set of Rabs tends to be universally conserved, and can coexist in different taxa with subfamily expansions and/or taxon- or species-specific Rabs. It is clear that no unique path to cellular complexity and specialisation exists, implying that any conclusion about the evolution of Rabs in a given taxon is not necessarily true for other eukaryotic taxa.

### Dating the origin of Rabs and expanding the LECA

**Figure 6:**
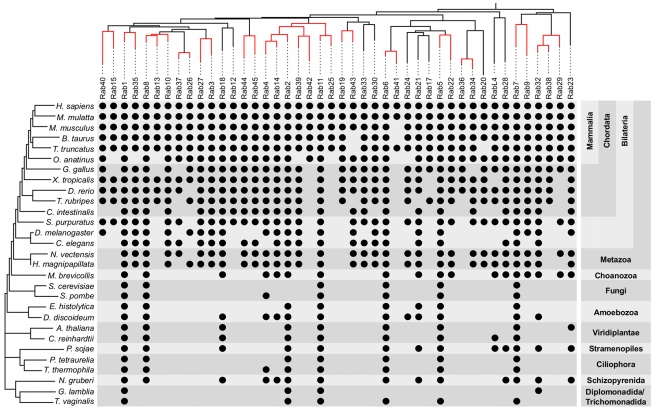
Phylogenetic profiles of human Rab subfamilies in selected organisms. A black dot reads as presence of the corresponding subfamily in the respective species. Rab subfamilies are ordered according to the top phylogenetic tree generated as explained in **Materials and Methods**. Branches with bootstrap support above 58 are coloured in red. The tree on the left represents the species' branching order and is derived from [115]–[118] together with the naming of the partially nested monophyletic groups on the right.

**Figure 7:**
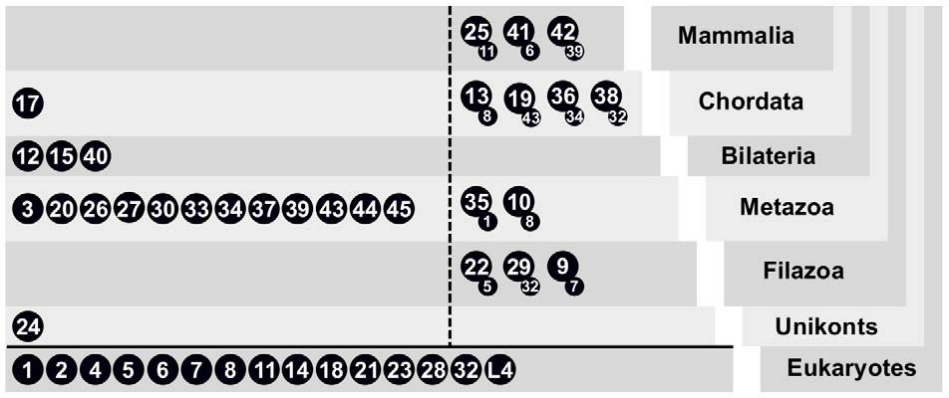
Summary of evolutionary age and duplication origin of human subfamilies. Each level represents a nested evolutionary stage from the LECA to humans (derived from [115], [119]) with one circle per human subfamily. Those subfamilies for which we could establish a clear origin, that is which subfamily it was derived from by duplication, are right from the dotted line with the subfamily it was derived from attached at the bottom right.

The systematic identification and classification of Rab repertoires in multiple branches of the eukaryotic tree of life allows the establishment of a phylogenetic profile for each Rab subfamily. As Metazoa and Fungi are the most extensively sampled and best annotated groups, we profiled human subfamilies (**Figure 6**) and determined their likely time of origin (**Figure 7**). For a detailed analysis of fungal Rabs see [26]. We further established the direction of duplication, *i.e.* from which Rab subfamily another emerged by duplication and subsequent divergence, by crossing their likely time of origin with a phylogenetic tree of the human Rab family. We reasoned that for two closely related Rabs, the one that is present in more taxa is likely the ancestral one. Since all Rabs are by definition paralogs and especially the deeper evolutionary relationships are unclear, we restricted the inference of direction of duplication to well supported branches. Here, we define well supported branches as those with bootstrap support higher than 58% in a tree of human Rabs, which is chosen to include the branch between Rab5 and Rab22 as their association is commonly accepted [47]–[51]. As further support, we note that all branches selected according to this criterion are also present in the tree of mouse Rabs we present below, however, in general 58% is not a strong branch support and should not be used indiscriminately on trees of other Rabs. Based on a 58% cutoff, one obtains directed duplication scenarios for a number of subfamilies as summarised in **Figure 7**. We term subfamilies with a clear origin as ‘derived’.

This analysis suggests new candidates for ancestral Rabs. Previously Rab1, 2, 4, 5, 6, 7, 8 and Rab11 [17], Rab18 [31], [52], Rab21 [30], [53] as well as Rab23 and 28 [32] could be mapped to more than one major branch of the eukaryotic tree, making them likely candidates to be present in the LECA. Our results support these assignments and reveal a new set of proteins that can be found in two or more basal eukaryotic taxa, namely Rab14, 32 and RabL4. Applying the same parsimony argument as previous studies suggests that these Rabs were part of the ancestral set of Rab in the LECA. Are these putative ancestral Rabs an artefact due to incorrect assignments or convergent evolution? We validated the automated subfamily classification by phylogenetic trees, and could not disprove their annotation (**Figures S4 A–C**). The possibility of convergent evolution is however harder to rule out. Regardless, an organism with 15 Rabs is not surprising and comparable with some unicellular eukaryotes [32], [33], and free living fungi frequently have less [26]. It is remarkable that with every new analysis the LECA appears to become increasingly more complex [54]. On functional grounds, mapping these Rabs to the LECA is plausible. RabL4, also known as IFT27, plays a role in ciliogenesis as part of the Intra Flagella Transport (IFT) machinery [55]. Flagella are believed to be ancestral characters, present in the LECA [56], [57]. Rab32 regulates transport to the pigmented/secretory granules [58], an animal-specific function, but it has also been claimed to have a mitochondria-related function [59], [60]. The known function of Rab14 in phagosome maturation and a recycling step at the TGN [61], [62] is less clearly ancestral, but it may lend support for a phagotrophic LECA as previously proposed [63].

In summary, our results support the claim that the LECA had a highly complex endomembrane system, and that secondary Rab losses have been dominant in the evolution of the major eukaryotic taxa [17].

### The Rab family in *Monosiga brevicollis* and the origins of animals

The emergence of multicellularity is one of the major transitions in evolution [64], which happened independently multiple times (see [65] for a recent review). There are several critical features necessary for the evolution of multicellular organisms, for example mechanisms for cell adhesion, cell polarity and inter-cellular communication. Little is known about how protein trafficking has evolved during this transition. We take advantage of our extensive annotation of the Rab family to derive the Rab complement prior to and after the emergence of multicellularity in Metazoa.

*Monosiga brevicollis* belongs to the Choanozoa, the closest unicellular relatives of Metazoa. The genome of this organism was only recently sequenced [66], and in the context of the validation of the Rabifier we conducted a detailed analysis of its Rab family. The phylogenetic tree in **Figure 2E** reveals a relatively large Rab family with nearly no subfamily expansions (see also **Figure 5**), *i.e.* mostly with a single member per subfamily (only Rab32 has two members). This is also observed in simpler animals like *D. melanogaster* and *C. elegans* [52], suggesting that larger subfamilies observed in mammals represent taxon-specific duplications. Secondly, we observe several organism-specific Rabs, which we labeled MbRabX. Consistent with results from the last section, the “invention” of new Rabs is a recurrent feature in multiple branches of the tree of life (*e.g.* [28], [30], [32], [52]). We observed the emergence of three novel sub-families, Rab9, 22, 29, none playing ‘animal-specific’ roles. The function of Rab29 is unknown, but Rab9 and Rab22 both appear to be involved in late endocytic traffic [49], [50], [67], [68]. Surprisingly, the genome of *M. brevicollis* codes for proteins previously believed to be specific to multicellular organisms, for example Cadherins [66], [69]. In animals, trafficking of the cell adhesion molecules Integrins and Cadherins is regulated by Rab4, 5, 11, 21 and 25 [70]–[73], and Rab5 and 7 [74], [75], respectively. Interestingly, these Rabs are also found in *M. brevicollis*, and—with the exception of Rab25—are all likely ancestral proteins. That highlights that complex new functions, as are for example the regulation of Cadherin and Integrin and ultimately cell adhesion, can be gained without inventing new subfamilies.

Our analysis revealed 14 Rab subfamilies that emerged at the base of Metazoa (**Figure 7**). Surveying the currently known functions of these animal-specific subfamilies suggests roles mainly in regulated secretion (Rab3 [76]–[79], Rab26 [80], Rab27 [79], [81]–[83], Rab33 [79], Rab37 [79], [84], Rab39 [85]), trafficking from (Rab10 [86]) and to the Golgi (Rab43 [87]) and more generally localisation at the Golgi (Rab30 [88]–[90], Rab33 [91], Rab34 [92], Rab43 [93]). Hence, our analysis suggests that the appearance of animals cooccurred with an important expansion and specialisation of the secretory pathway.

### A model for Rab subfamily innovation

Gene duplication is a frequent mode of gene gain in eukaryotes. This is well illustrated by the expansion of the Rab family in emergence and evolution of Metazoa. Following gene duplication, the most common fate for one of the duplicates is accumulation of mutations up to the point of pseudogenisation. In the alternative case, the retention of both duplicates has been explained by different theoretical scenarios, recently surveyed in [94]. Most prominently, either divergence results in gain of a beneficial new function (neo-functionalisation) by one of the duplicates, or disruption of complementary parts of the function in each of the genes leaves both paralogs indispensable to perform the original function (sub-functionalisation). As discussed in [94], those models predict distinct types and strengths of selective forces acting on the two duplicates allowing to test and distinguish amongst putative scenarios. Namely, while in both neo- and subfunctionalisation the new copy indistinguishably evolves neutrally, detecting purifying selection acting on the original copy is an indication of neo-functionalisation, whereas relaxed purifying or neutral selection is suggestive for sub-functionalisation. In the case of Rabs, **Figure 6** shows that the original copy is conserved and keeps its identity as the original subfamily, whereas the new copy initiates a distinct subfamily defined by a discernible level of sequence divergence. We interpret this pattern as evidence that the mode by which the Metazoan Rab family expands is most probably neo-functionalisation rather than subfunctionalisation.

**Figure 8:**
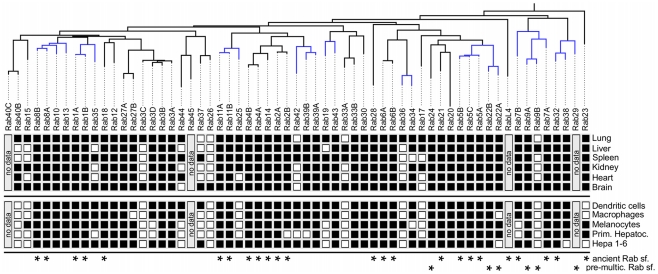
Increasing tissue specificity in expression of derived Rabs in mice. Summary of PCR experiments establishing expression (black squares) or lack thereof (white squares) of mouse Rabs in six tissues and five mouse cell lines. Stars on the bottom indicate subfamilies which we found already present in LECA, and that predate the evolution of multicellularity (see **Figure 7**). Branches coloured in blue in the phylogenetic tree of mouse Rabs on the left are those for which we test the hypothesis that derived subfamilies are expressed in the same or in a subset of tissues of the Rab they were derived from (see **Figure 7** for a summary of which Rabs have a clear origin). Abbreviations: subfamily (sf.), primary Hepatocytes (Prim. Hepatoc.), multicellularity (multic.), last eukaryotic common ancestor (LECA).

To gain further insights into the nature of the gain of function, we asked whether the derived Rab subfamilies show differences in tissue-specificity that could hint at the type of newly evolved functions. To this end, we investigated tissue-specificity in expression of Rabs in mouse tissues and cell lines (**Figure 8**) by means of PCR (see **Materials and Methods**). We also analysed publicly available microarrays (**Figures S5, S6**) which overall corroborate the trends described in the following.

First, we observed that all ancestral Rabs are widely expressed (*i.e.* in all tested tissues), most probably performing general functions required in all tissues. Similarly, Rabs that predate the advent of multicellularity are also broadly expressed, a general phenomenon that has been described for genes which emerged prior to multicellularity [95]. Second, for the derived subfamilies in which a clear directionality of duplication could be established (see **Figure 7**), we detected a trend for an increase in tissue specificity, *i.e.* a reduction in number of tissues in which the Rab is expressed relative to its progenitor subfamily. For example, Rab34 is expressed in all tissues investigated but the liver, whereas the derived Rab36 is only expressed in lung and brain. Thirdly, at no time we observe complementary expression, *i.e.* a pair of subfamilies which have opposite tissue specificities.

Overall, these observations are strong indications that derived subfamilies are retained for a new tissue-specific functions, different from or at least complementing the progenitor ones. Thus, our results support a neo-functionalisation model explaining the retention of novel Rab sub-families in Metazoa. This model makes several predictions about expression patterns of Metazoan Rabs for which we could not derive expression data. Concretely, Rab41 which we only find in primates and dolphin is expected to show a restricted tissue expression, as its origin from Rab6 is statistically well supported. Rab29 is expected to be ubiquitously expressed despite its clear origin from Rab32 as it predates the evolution of multicellularity, a prediction at least supported by our microarray-based analysis (**Figure S5**).

One notable observation is that the tested mouse tissues express an unexpectedly high number of distinct Rabs. This is also observed in individual cell lines, which indicates that it is not an artefact from multiple cell types mixed in the tissue. While it is clear that Rabs are expressed at different levels [96] (see also **Figure S6**), our results from a more sensitive method than microarrays reveal that the tissue-specific Rabs may be more widely expressed than previously anticipated. It remains to be investigated whether the low levels of expression we can detect by PCR are functionally significant.

### Conclusions

We developed the ‘Rabifier’, a bioinformatics tool to identify and classify Rabs from any set of protein sequences with no need for additional phylogenetic information, which we make available as a web tool for the community. We deployed the Rabifier on 247 proteomes predicted from complete genome sequences, generating the first comprehensive view of the Rab sequence space, which we also make available in form of a browsable database of Rab proteins. We envisage that cell biologists interested in specific organisms may use RabDB and the Rabifier as a first description of the family, at accuracy levels we showed to be very high. In fact, our predictions are well suited to be the first step towards high quality manual annotations. Furthermore, we introduced unified and objective criteria for the annotation of Rabs which is especially important for large-scale comparative studies, which can now be grounded on a coherent body of data.

The classification of Rab repertoires in hundreds of genomes gives us the first global view of the Rab family in evolution, revealing that this family followed different routes in each branch of the tree. Massive expansions co-exist with extensive losses. These expansions can vary from taxon to taxon, suggesting that care must be taken when transferring information amongst different branches of the tree of life. In this respect, future work may focus on understanding the detailed evolutionary patterns in eukaryotic taxa other than Metazoa, which we analysed here. It appears that plants are ideal candidates for such a study as multiple genomes have been sequenced covering both unicellular and multicellular organisms.

One of the perhaps most surprising observations we made was the extension of RabX's, *i.e.* Rabs that cannot be assigned to any previously characterised subfamily. Hence, a major bioinformatic and cell biological challenge now is to identify how many Rab subfamilies exist overall, and to establish their conservation or taxon-specificity. Here, we started this classification by proposing new Rab subfamilies derived from clustering of RabX's with respect to their sequence similarity. We hope to stimulate further research which may allow the refinement of our criteria and ultimately the definition of a Rab subfamily. The notion of Rab subfamily is supposed to reflect both evolutionary history and functional information, but has historically been mixed with less clear criteria. In the absence of functional information for all Rabs, phylogenetic analysis becomes particularly important, especially for functional prediction. In this context, it is all the more serious that we found a notorious frailty of Rab trees. Factors such as choice of sequences, outgroups, alignment program, probabilistic model and program implementing it contribute to very different trees (compare for example [52], [97], [98] and **Figures S4 A–C**). We thus need to derive objective criteria that define a Rab subfamily which go beyond the clearly outdated yet still useful sequence identity cutoff [24]. Possibilities are for example to introduce soft thresholds depending on background divergence levels within a given taxon, or to restrain the area considered to measure sequence divergence to the functionally relevant regions.

We focused on the evolutionary path from the LECA to mammals in order to gain insight into the mechanism of functional innovation within the Rab family. Based on objective and re-usable criteria we were able to map directionality to duplications clarifying the origin of some human subfamilies. Crossing these relations with data on tissue-expression patterns of Rab genes, we proposed that neo-functionalisation best explains the emergence of new subfamilies. More recent subfamilies are most likely retained for newly evolved tissue-specific functions and coexist with older ones in a subset of tissues. It remains to be determined whether the same happens within a subfamily, *i.e.* whether a RabXa and a RabXb represent cases of neo- or sub-functionalisation [99]. This is particularly relevant to conceptually tell apart isoforms and distinct subfamilies. As we restricted our analysis to subfamilies present in humans, it is important now to test whether the same neo-functionalisation scenario is observed in other branches of the tree of life. As mentioned before, plants appear to be ideal candidates to extend this analysis. Finally, while we studied the fate of new subfamilies in the context of tissue-specific expression, it will be important to understand the contribution of subcellular re-localisation to neo-functionalisation [100], [101].

New generations of sequencing methods promise to change that scale at which we perform comparative analysis in cell biology. But for this change to reach the cell biology community, we need the appropriate tools that allow the non-bioinformatician to take advantage of all the emerging data. The Rabifier is one such tool, tailored to enable the cell biologist to analyse protein repertoires in hundreds of genomes.

## Materials and Methods

### Ethics statement

C57BL/6 mice were bred and housed in the pathogen-free facilities of the Instituto de Gulbenkian de Ciência (IGC). Mouse experimental protocols were approved by the Institutional Ethical Committee and the Portuguese Veterinary General Division.

### The set of human Rabs

Before we devised a workflow able to identify and classify Rabs, we decided which protein subfamilies we considered being human Rab subfamilies. Since the early genomic analyses of the human Rab repertoire reporting subfamilies 1 to 40 (with exception of 16) [24], five subfamilies have been newly discovered (41 to 45/Rasef) [102]. Besides those clear cases, the distinction remained less obvious for those which are termed ‘Ran’ and ‘Rab-like’, each of which we briefly discuss in the following.

Rans control nucleocytoplasmic shuttling [103], and are frequently considered to be members of the Rab family [97], [102]. This view is supported by our own phylogenetic analysis (see tree in **Figure S3**), although without strong bootstrap support. Due to the distinct function and localisation [103] partly within the nucleus we do not further consider Rans in our dataset. However, Rans have recently been linked to ciliary entry of certain kinesins [104], and they may be included in the future.

RabL2 proteins were already mentioned in [24] where it is concluded that they are not Rabs, amongst others due to non-conforming RabF motifs. In [97], RabL2's are said to cluster together with Rans, which we do not include in our analysis. The tree of human GTPases shown in [98] suggests that RabL2 proteins branch of Rhos at an early stage. Finally, our own tree of human GTPases (**Figure S3**) positions RabL2s at the periphery of the Rab branch, yet with little bootstrap support. Altogether, we do not see enough evidence for RabL2 proteins to be considered Rabs. The situation is similar for RabL3 and RabL5. Colicelli clusters them together with Rans [97], whereas in [98] both reside on a branch with Arfs though classified as belonging to none of the classes Rab, Ras, Arf, Rho or Ran. Our tree of human GTPases suggests that RabL5 and Arfs have a common ancestor, equally so RabL3 and RabL2, hence we ignored both in our further analysis. Rab7L1 is nearly identical to Rab29 and represents a simple case of naming ambiguity, as has already been pointed out in [24].

The last case is RabL4, which all [97], [98], [102] consider being a Rab. We confirmed that interpretation by detecting and validating four RabF motifs, as well as by our phylogenetic tree, which places RabL4 within Rabs. However, we only group RabL4 together with Rab28 as suggested in [97], [102] when no GTPase other than the human Rab subfamilies 1 to 45 are included (see trees in **Figure S3** and **Figures S4 A–B**). In mouse, RabL4 is not classified as being monophyletic with Rab28 (see **Figure S4 C**).

### The Rabifier

We give some technical details about the implementation of the Rabifier which for the sake of brevity have been omitted above. For information on the computation of the confidence scores see **Text S1**.

In the first phase (**Figure 1A**), the profile HMM's representing the G-protein family domain are either run manually using Perl scripts (as of June 2010) provided by Superfamily [36] and HMMER 2.3.2 [35], or in the case the sequences have been retrieved from the Superfamily database [41] the domain structure is taken directly from Superfamily. Note that Superfamily is a pure protein resource that contains proteomes predicted from genome sequences. It does not provide information about the underlying genes systematically, hence counts of how many Rab genes are present in a specific genome can generally not be derived from Superfamily. BLASTp [37] queries are performed with soft masking (parameters -F m S) and considered up to an e-value threshold of 10^−10^. Our reference set of sequences not being Rabs is provided as **Dataset S1**, whereas the reference database of Rabs are the sequences accessible at www.RabDB.org with redundancy removed using CDHit (at a 90% sequence identity threshold) [105]. Our reference data set of Rabs covers more than just the human subfamilies, namely previously published and functionally described subfamilies from *Arabidopsis thaliana* (AtRabA1, AtRabA3–AtRabA6, AtRabC2, AtRabD1, AtRabF1, AtRabG1) [31], yeast (yptA, ypt10, ypt11), *Drosophila melanogaster* (DmRabX1–DmRabX6, DmRab9D, DmRab9F) and *C. elegans* (CeRabY6) [52]. Furthermore, as detailed in the main text we proposed a set of hypothetical subfamilies which we integrated into our reference set. The members and phylogenetic distribution of these hypothetical subfamilies can browsed directly on our web site www.RabDB.org. The last stage of the first phase is performed using the Motif Alignment & Search Tool (MAST) (motif finding threshold 0.0005) [106] from the MEME-suite [107], with probabilistic representations of the motifs ‘igvdf’, ‘klqiw’, ‘rfxxxt’, ‘yyrga’, ‘lvydit’ [24] as input generated on our reference database of Rabs beforehand using MEME.

In the second phase (**Figure 1B**), RPS-BLAST queries [38] are performed with standard parameters and an e-value threshold of 10^−5^, with position-specific scoring matrices (PSSM) previously generated by Ψ-BLAST on all members of each of the Rab subfamilies present in our reference database.

### Hypothetical subfamilies

The hypothetical subfamilies result from two distinct clustering steps. First, we clustered sequences classified as RabX by the Rabifier and belonging to the same genome at a sequence identity threshold of 70% [24]. In order to resolve the potential conflicts caused by sequences that belong to several clusters at the same time, we applied MCL [108] (inflation parameter 2.0), which resulted in a clean partition, *i.e.* non-overlapping clustering, of the sequences. In a second step, we merged the resulting clusters across genomes if at least one pair of sequences across clusters shared a sequence identity over 70%. We chose this threshold as it is the lowest which ensures meaningful clusters, that is clusters which in their majority respect taxa boundaries.

### Phylogenetic trees

All phylogenetic trees of Rabs and GTPases presented in this article have been generated with PhyML [109], which implements a Maximum Likelihood probabilistic model, using standard parameters and 100 bootstraps. Alignments were performed with MAFFT [110], and manually edited to remove sites with deletions using Jalview [111]. The human trees have been generated using human kRas as an outgroup, the mouse trees using mouse kRas as outgroup, and the mixed tree of human and *Monosiga brevicollis* Rabs uses both human and *M. brevicollis* kRas as outgroups. Sequence accessions of all sequences can be taken from **Table S2**. Tree visualisations have been generated with Figtree (http://tree.bio.ed.ac.uk/software/figtree/). The tree of human Rabs not displaying isoforms (see **Figure 5** **,** **Figure 6**) has been generated by removing isoforms and keeping the longest branch as representative of the corresponding subfamily.

### Rab PCR of mouse organs and cells

#### Cell lines and primary cells

We decided to use both cell lines and primary cells. Cell lines are populations of cells that grow and replicate continuously, *i.e.* that have undergone genetic transformations which result in indefinite growth potential. They are prone to genotypic and phenotypic drifting, and can both lose tissue-specific functions and acquire a molecular phenotype quite different from primary cells. In contrast to that, primary cells have a finite lifespan but reflect the *in vivo* situation, despite their added complexity. In the following, we list the protocols we followed to obtain our cell material.

Mouse hepatoma Hepa 1–6 cells were cultured in DMEM supplemented with 10% FCS, 100 U/ml penicillin and 100 µg/ml streptomycin, maintained at 37°C in 10% CO_2_ until the cells were 80% confluent and then used to extract RNA. The melanocyte cell line melan-ink was cultured in RPMI 1640 with glutamax and hepes, supplemented with 10% FCS, 0.1 mM 2-mercaptoethanol, 200 nM phorbol 12-myristate 13-acetate, 100 U/ml penicillin and 100 µg/ml streptomycin at 37°C with 5% CO_2_. We extracted RNA when the cells were 80% confluent. Primary dendritic cells (DC) were isolated from the bone marrow of C57BL/6 mice. Femurs and tibia were removed, both ends of the bones cut and the bone marrow flushed using a syringe. Cells were cultured in plates (2–4×10^6^ cells per plate) with 10 ml of Iscove's medium with glutamax and hepes, supplemented with 10% FCS, 100 U/ml of penicillin, 100 µg/ml streptomycin, 5×10^−5^ M 2-mercaptoethanol, 0.5 mM sodium pyruvate, containing 2% of culture supernatant from X63/0 myeloma cells transfected with mouse GM-CSF cDNA. After 3 days of culture, new medium with GM-CSF was added to each plate. After 7 days of culture, the non-adherent cells were collected and processed for purification with magnetic beads on MACS columns (Miltenyi Biotec). Cells were incubated with CD11c^+^ magnetic beads and passed through the column. The positively selected cells were pelleted by centrifugation for RNA extraction. Typically more than 90% of the positive cell population expressed the dendritic cell marker CD11c^+^ as determined by flow cytometry. Primary macrophages were isolated from the bone marrow of C57BL/6 mice using the same procedure as for the DC and matured in M-CSF-containing media. Cells were cultured in plates (4×10^6^ cells per plate) with 10 ml of Iscove's medium containing 30% of L929 cell-conditioned media as a source of M-CSF. After 4 days of culture, additional media with M-CSF was added. Macrophages were used after 8 days in culture for RNA extraction after removing non-adherent cells. Typically more than 90% of the cell population expressed the macrophage marker CD11b (Mac-1) as determined by flow cytometry. Primary hepatocytes were obtained from C57BL/6 mice as previously described in [112] and used to extract RNA.

#### RNA isolation and cDNA synthesis

Tissue samples (Spleen, Liver, Kidney, Brain, Heart and Lung) were rapidly dissected and immediately homogenised in Trizol reagent. Total RNA was purified from the cells or tissues using a RNeasy Mini Kit (Qiagen) following the manufacturer's instructions. For cDNA synthesis 500 ng of total RNA was reverse transcribed using the “First-Strand cDNA synthesis kit” (Roche) following the manufacturer's instructions.

#### PCR and DNA analysis of Rab GTPase expression profiles

PCR was performed on the cDNA product to assess the expression of Rab GTPases. The primers used for amplification can be taken from **Table S3**. The PCR amplification was performed in a reaction mixture containing 1× green Go Taq buffer (Promega), 1 mM MgCl_2_, 0.2 mM of dNTP mix, 2.5 U of Taq polymerase (Promega) and specific primers at a final concentration of 0.5 µM, followed by a denaturation step of 3 min at 94°C and a 32-cycle program consisting of 94°C for 40 s, 58°C for 40 s and 72°C for 1 min. The final amplification mixture was separated in 1.2% agarose gel containing ethidium bromide and photographed under UV illumination.

## Supporting Information

Dataset S1 **Rabifier's negative reference sequences.**
(FASTA)

Figure S1 **Sequence identity to best hit within same subfamily.** Histogram of sequence identity of all sequences in our reference database to their respective best hit within the same subfamily (itself excluded). Subfamilies can contain sequences from organisms anywhere in the eukaryotic tree. The threshold is the minimal required identity for a sequence to be attributed to the subfamily of its best hit (see **Figure 1**). It is chosen to minimise the number of times a sequence is annotated as belonging to the unspecified subfamily RabX although it is a member of the same subfamily as its best hit.
(PDF)

Figure S2 **Linear regression of number of Rabs against genome size.** Data consists of the 247 genomes profiled by the Rabifier. The taxa are shown in different colours.
(PDF)

Figure S3 **Phylogenetic tree of some human small GTPases.** All bootstrap values are included. For information on how the tree has been generated check **Materials and Methods** in the main article. All sequence accessions are listed in **Table S2**. The representation has been generated with Dendroscope [120].
(PDF)

Figure S4 **Phylogenetic trees of some Rab subfamilies.** Panel (**A**) contains Rab subfamilies Rab14, 4, 2, panel (**B**) Rab32, 7, 23 and finally panel (**C**) RabL4, 28. Each of the trees covers different taxa. For information on how the trees have been generated check **Materials and Methods** in the main article. All sequence accessions are listed in **Table S2**. All representations have been generated with Dendroscope [120]. Abbreviations: *Homo sapiens* (Hs), *Mus musculus* (Mm), *Monosiga brevicollis* (Mb), *Naegleria gruberi* (Ng), *Leishmania major* (Lm), *Leishmania braziliensis* (Lb), *Leishmania infantum* (Li), *Trypanosoma brucei* (Tb), *Trypanosoma cruzi* (Tc), *Plasmodium falciparum* (Pf), *Toxoplasma gondii* (Tg), *Tetrahymena thermophila* (Tt), *Paramecium tetraaurelia* (Pt), *Giardia lamblia* (Gl), *Trichomonas vaginalis* (Tv), *Phytophtora infestans* (Pi), *Phytophtora sojae* (Ps), *Micromonas pusilla* (Mp), *Volvox carteri* (Vc).
(PDF)

Figure S5 **Tissue specificity in expression of mouse Rabs.** We analysed microarray data from the Mouse Exon Atlas (GEO accession: GSE15998) generated on an Affymetrix Mouse Exon 1.0 ST Array (GEO accession: GPL6193) downloaded from [121]. We mapped probes to genes using the R package provided in [122]. The data analysis has been performed in R using Bioconductor's ‘affy’ library. After applying RMA [123], we combined biological replicates by averaging their expression value. To transform continuous expression (**B**) into a discrete present/absent pattern (**A**) we chose a threshold (5.0) that maximises the agreement with the PCR data from **Figure 8** while achieving a balanced number of false positives (28) and false negatives (25).
(PDF)

Figure S6 **Quantitative expression of Rabs in mouse tissues.** Figure (**A**) plots the same data as shown in **Figure S5** prior to the binarisation via thresholding. (**B**) shows the average expression across the mouse tissues (cell lines not included).
(PDF)

Figure S7 **Distribution of Rabs belonging to non-human subfamilies.** The histogram details for each taxon how we classified those Rabs not belonging to human subfamilies. Subfamilies falling into the orange category have been previously described in the literature, whereas all other subfamilies result from clustering of the sequences as described in **Materials and Methods**. See **Figure 4** for an overview of the number of subfamilies in each category.
(PDF)

Table S1 **Results of Rabifier validation.** We list the accessions of the proteins used to perform the validations in **Figure 2**. All Superfamily accessions refer to the release as of September 2009. As indicated in the upper table, four sequences from [32] have invalid IDs and are replaced by sequences with the same annotation from the newest release of the Trypansoma genome project (ftp://ftp.sanger.ac.uk/pub/databases/T.brucei_sequences/T.brucei_genome_v4/). Sequences missing either type of accession are those unique either to the respective paper or our automated scan of the full genome using the Rabifier. For the latter, we manually checked the sequences for being false positives, however, as we could not recover the full genomes used in the initial studies and the protein sequences predicted from fully sequenced genomes are not stable we expected certain amounts of discrepancies. In the last part of the table containing the results of the alternative strategy for *M. brevicollis*, ‘NOT A RAB’ stands for total lack of information allowing to infer that the protein may be a Rab, ‘RAB’ simply stands for lack of any subfamily annotation. The regular expression used to automatically scan the ‘region’ annotation for family- and subfamily-information was ‘(?:∧|\s)([\w]{2}rab{1}\w?\d{1,2}\w?)|(rab{1}\w?\d{1,2}\w?)|(rab{1})’.
(PDF)

Table S2 **Accessions of all sequences.** Uniprot [124] accessions of all sequences used to generate the phylogenetic trees in **Figures 4** **–** **7** and **Figures S3, S4**. The Uniprot human Rab42 sequence is most probably only a fragment, hence in all cases the alternative sequence from Superfamily [41] is used. In case of multidomain proteins (human and mouse Rab44 and Rab45), alignments were generated only using the designated residues. Isoforms of the same Rab subfamily are distinguished by prime symbols. Abbreviations: *Homo sapiens* (Hs), *Mus musculus* (Mm), *Monosiga brevicollis* (Mb), *Naegleria Gruberi* (Ng), *Leishmania major* (Lm), *Leishmania braziliensis* (Lb), *Leishmania infantum* (Li), *Trypanosoma brucei* (Tb), *Trypanosoma cruzi* (Tc), *Plasmodium falciparum* (Pf), *Toxoplasma gondii* (Tg), *Tetrahymena thermophila* (Tt), *Paramecium tetraaurelia* (Pt), *Giardia lamblia* (Gl), *Trichomonas vaginalis* (Tv), *Phytophtora infestans* (Pi), *Phytophtora sojae* (Ps), *Micromonas pusilla* (Mp), *Volvox carteri* (Vc).
(PDF)

Table S3 **Primer sequences used to characterise mouse Rabs.**
(PDF)

Text S1 **Describes how the Rabifier computes Rab family and subfamily scores.**
(PDF)
