# Supplementary material for: Thousands of Rab GTPases for the Cell Biologist
Source: PLoS Comput Biol. 2011 Oct 13;7(10):e1002217. doi: 10.1371/journal.pcbi.1002217 (PMC3192815; doi:10.1371/journal.pcbi.1002217)
Supplement: Figure S1 — Sequence identity to best hit within same subfamily. Histogram of sequence identity of all sequences in our reference database to their respective best hit within the same subfamily (itself excluded). Subfamilies can contain sequences from organisms anywhere in the eukaryotic tree. The threshold is the minimal required identity for a sequence to be attributed to the subfamily of its best hit (see Figure 1 ). It is chosen to minimise the number of times a sequence is annotated as belonging to the unspecified subfamily RabX although it is a member of the same subfamily as its best hit. (PDF) [file pcbi.1002217.s002.pdf]

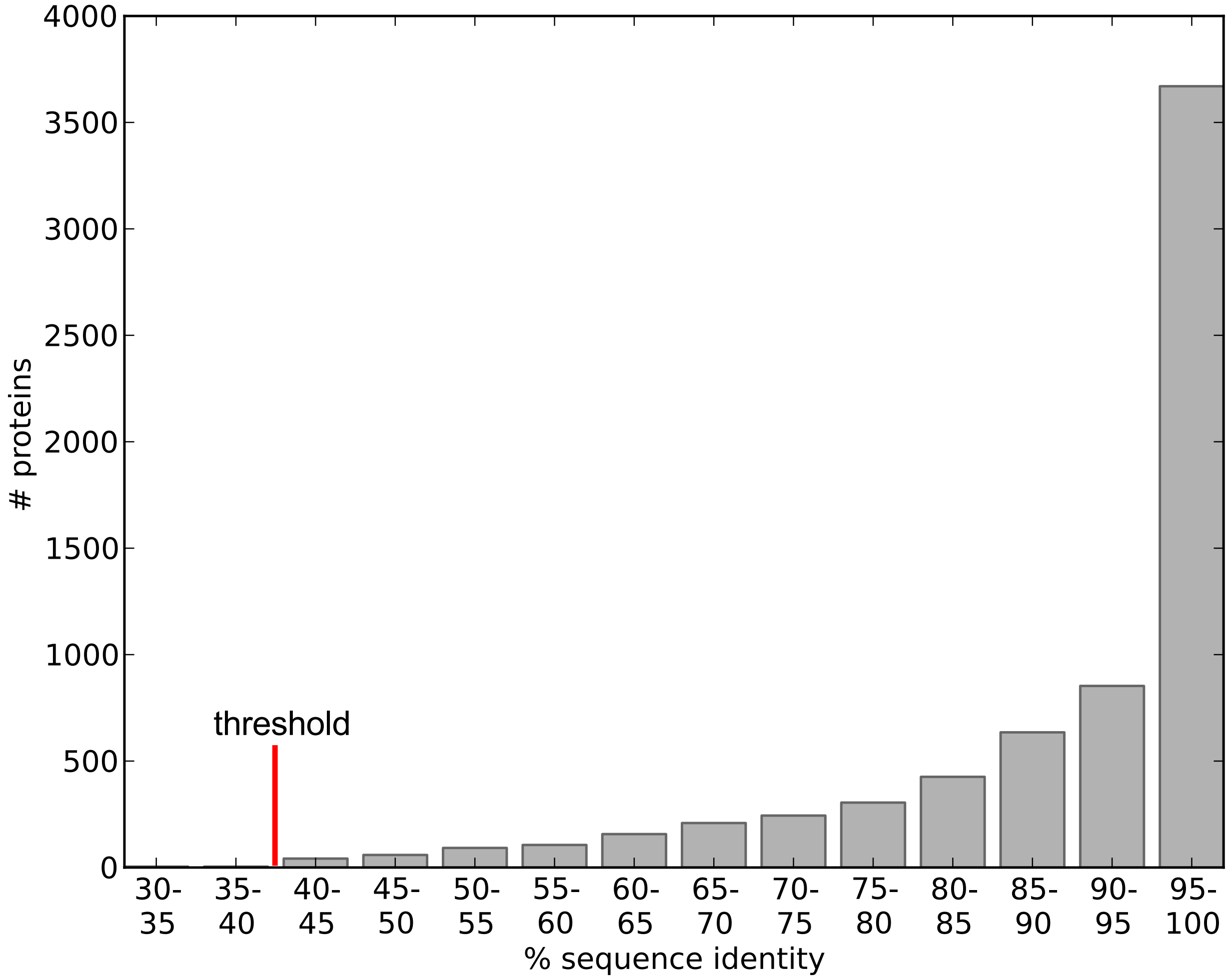

Figure S1. Sequence identity to best hit within same subfamily. Histogram of sequence identity of all sequences in our reference database to their respective best hit within the same subfamily (itself excluded). Subfamilies can contain sequences from organisms anywhere in the eukaryotic tree. The threshold is the minimal required identity for a sequence to be attributed to the subfamily of its best hit (see **Figure 1**). It is chosen to minimise the number of times a sequence is annotated as belonging to the unspecified subfamily RabX although it is a member of the same subfamily as its best hit.
