# Supplementary material for: Thousands of Rab GTPases for the Cell Biologist
Source: PLoS Comput Biol. 2011 Oct 13;7(10):e1002217. doi: 10.1371/journal.pcbi.1002217 (PMC3192815; doi:10.1371/journal.pcbi.1002217)
Supplement: Figure S2 — Linear regression of number of Rabs against genome size. Data consists of the 247 genomes profiled by the Rabifier. The taxa are shown in different colours. (PDF) [file pcbi.1002217.s003.pdf]

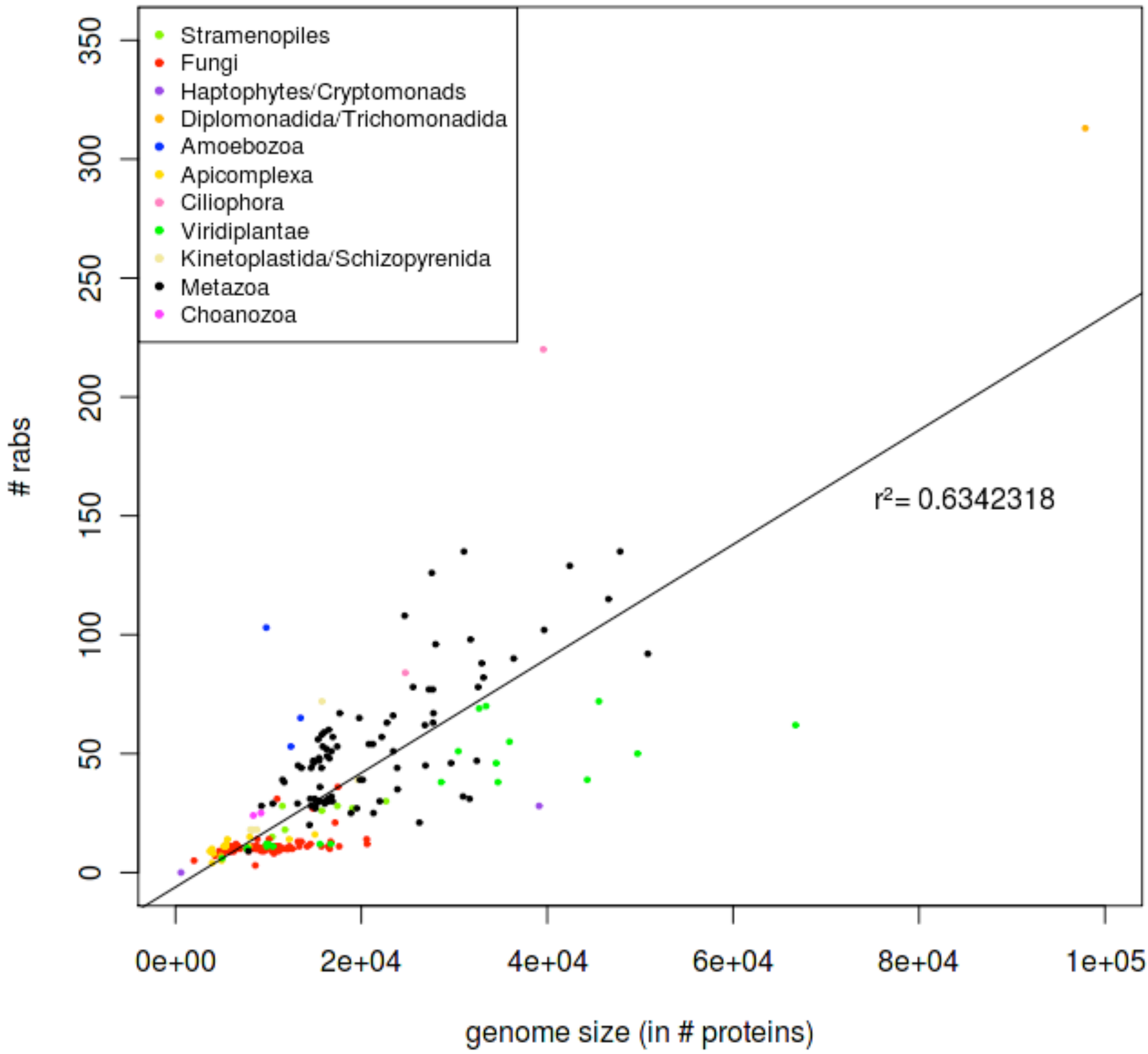

Figure S2. Linear regression of number of Rabs against genome size. Data consists of the 247 genomes profiled by the Rabifier. The taxa are shown in different colours.
