# Supplementary material for: Thousands of Rab GTPases for the Cell Biologist
Source: PLoS Comput Biol. 2011 Oct 13;7(10):e1002217. doi: 10.1371/journal.pcbi.1002217 (PMC3192815; doi:10.1371/journal.pcbi.1002217)
Supplement: Figure S3 — Phylogenetic tree of some human small GTPases. All bootstrap values are included. For information on how the tree has been generated check Materials and Methods in the main article. All sequence accessions are listed in Table S2. The representation has been generated with Dendroscope [120]. (PDF) [file pcbi.1002217.s004.pdf]

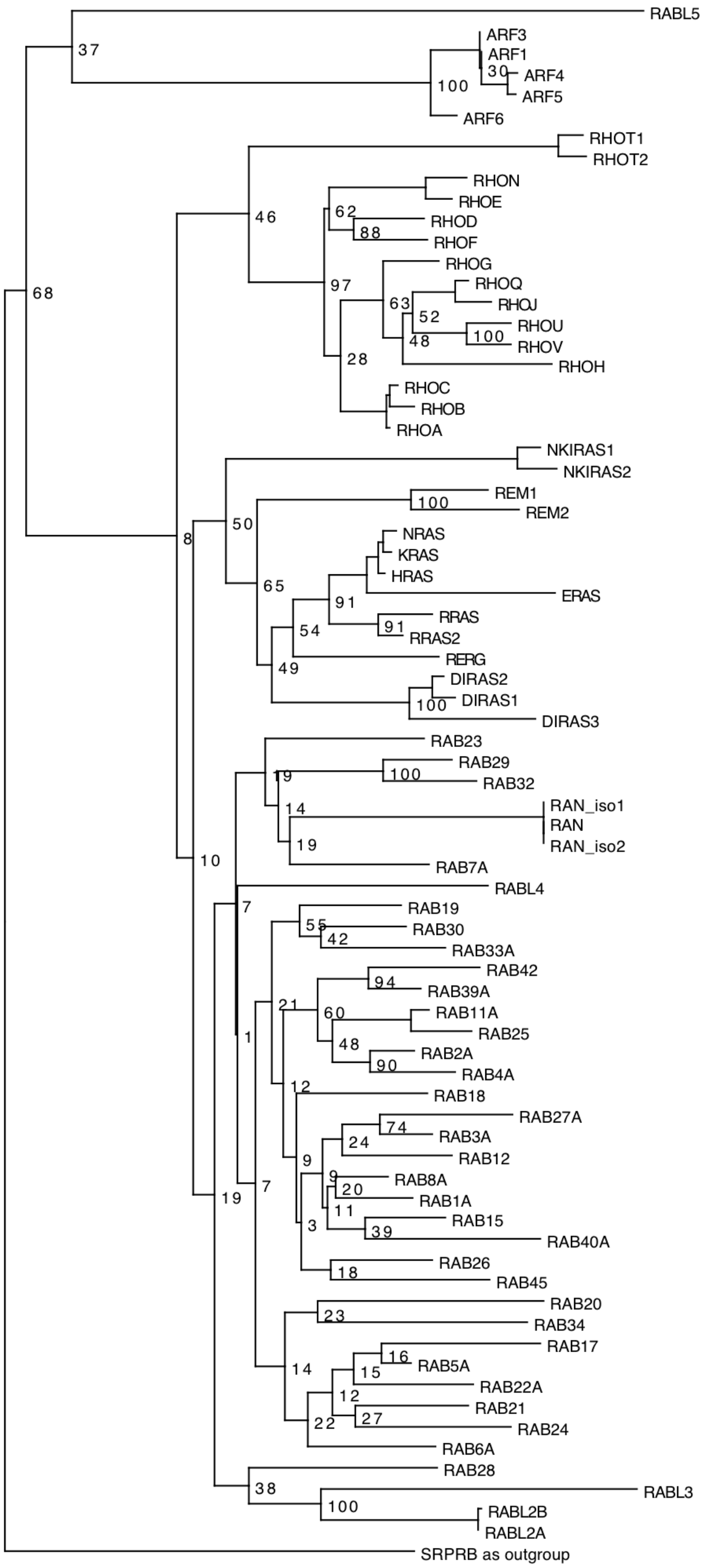

Figure S3. Phylogenetic tree of some human small GTPases. All bootstrap values are included. For information on how the tree has been generated check **Materials and Methods** in

the main article. All sequence accessions are listed in **Table S2**. The representation has been generated with Dendroscope [1].

References

1. Huson DH, Richter DC, Rausch C, Dezulian T, Franz M et al. (2007) Dendroscope: An interactive viewer for large phylogenetic trees. BMC Bioinformatics 8: 460.
