# Supplementary material for: Thousands of Rab GTPases for the Cell Biologist
Source: PLoS Comput Biol. 2011 Oct 13;7(10):e1002217. doi: 10.1371/journal.pcbi.1002217 (PMC3192815; doi:10.1371/journal.pcbi.1002217)
Supplement: Figure S4 — Phylogenetic trees of some Rab subfamilies. Panel (A) contains Rab subfamilies Rab14, 4, 2, panel (B) Rab32, 7, 23 and finally panel (C) RabL4, 28. Each of the trees covers different taxa. For information on how the trees have been generated check Materials and Methods in the main article. All sequence accessions are listed in Table S2. All representations have been generated with Dendroscope [120]. Abbreviations: Homo sapiens (Hs), Mus musculus (Mm), Monosiga brevicollis (Mb), Naegleria gruberi (Ng), Leishmania major (Lm), Leishmania braziliensis (Lb), Leishmania infantum (Li), Trypanosoma brucei (Tb), Trypanosoma cruzi (Tc), Plasmodium falciparum (Pf), Toxoplasma gondii (Tg), Tetrahymena thermophila (Tt), Paramecium tetraaurelia (Pt), Giardia lamblia (Gl), Trichomonas vaginalis (Tv), Phytophtora infestans (Pi), Phytophtora sojae (Ps), Micromonas pusilla (Mp), Volvox carteri (Vc). (PDF) [file pcbi.1002217.s005.pdf]

(A)

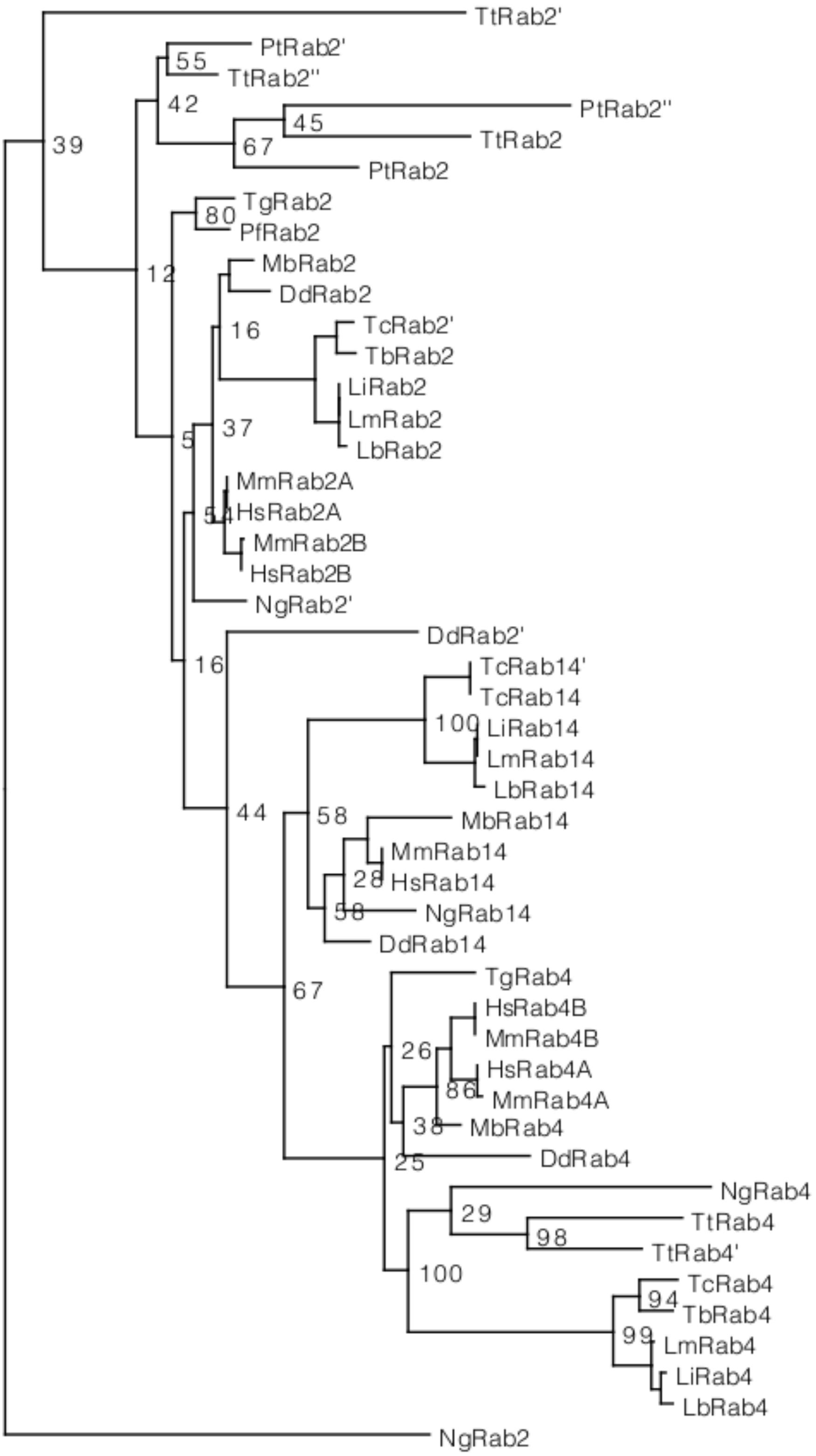

TtRab2'
PtRab2'
55
TtRab2''
42
PtRab2''
45
TtRab2
39
67
PtRab2
TgRab2
80
PfRab2
12
MbRab2
DdRab2
16
TcRab2'
TbRab2
LiRab2
37
LmRab2
LbRab2
MmRab2A
HsRab2A
MmRab2B
HsRab2B
NgRab2'
DdRab2'
16
TcRab14'
TcRab14
100
LiRab14
LmRab14
LbRab14
44
58
MbRab14
MmRab14
28
HsRab14
NgRab14
DdRab14
TgRab4
67
HsRab4B
MmRab4B
26
HsRab4A
86
MmRab4A
38
MbRab4
25
DdRab4
NgRab4
29
TtRab4
98
TtRab4'
100
TcRab4
94
TbRab4
99
LmRab4
LiRab4
LbRab4
NgRab2

(B)

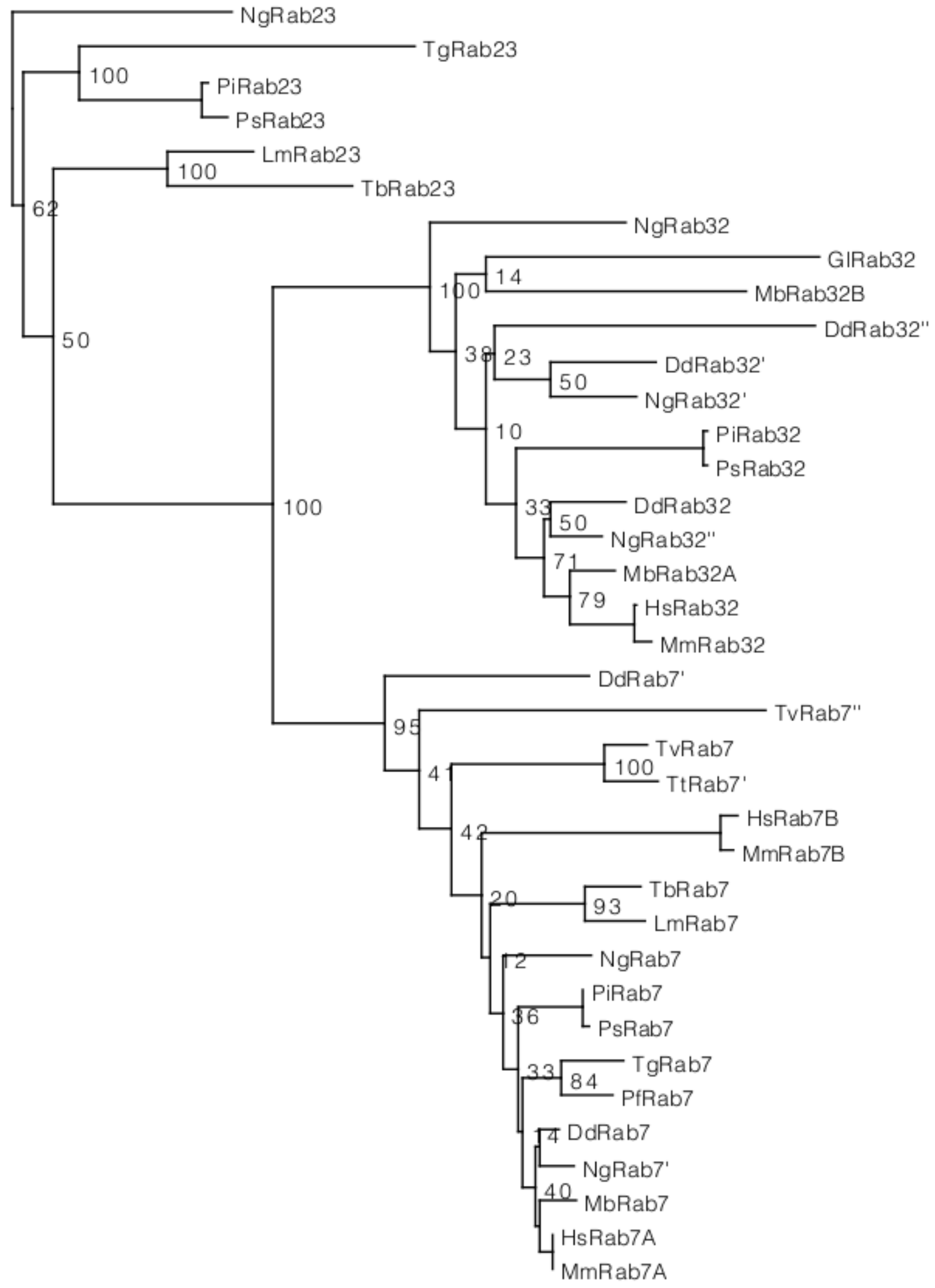

NgRab23
TgRab23
100
PiRab23
PsRab23
LmRab23
100
TbRab23
62
NgRab32
GlRab32
14
100
MbRab32B
DdRab32''
38
23
DdRab32'
50
50
NgRab32'
10
PiRab32
PsRab32
100
33
DdRab32
50
NgRab32''
71
MbRab32A
79
HsRab32
MmRab32
DdRab7'
TvRab7''
95
TvRab7
100
41
TtRab7'
HsRab7B
42
MmRab7B
20
TbRab7
93
LmRab7
12
NgRab7
PiRab7
36
PsRab7
33
TgRab7
84
PfRab7
4
DdRab7
NgRab7'
40
MbRab7
HsRab7A
MmRab7A

(C)

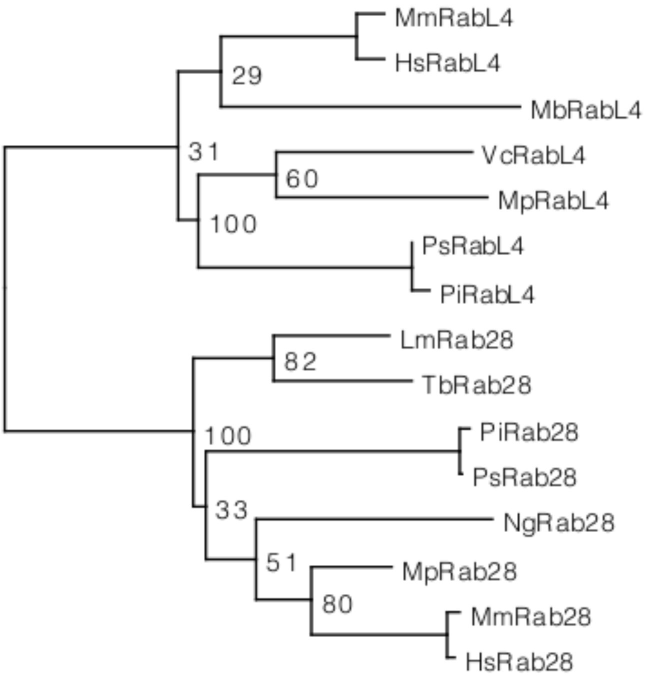

Figure S4. Phylogenetic trees of some Rab subfamilies. Panel (**A**) contains Rab subfamilies Rab14, 4, 2, panel (**B**) Rab32, 7, 23 and finally panel (**C**) RabL4, 28. Each of the trees covers different taxa. For information on how the trees have been generated check **Materials and Methods** in the main article. All sequence accessions are listed in **Table S2**. All representations have been generated with Dendroscope [1].

Abbreviations: *Homo sapiens* (Hs), *Mus musculus* (Mm), *Monosiga brevicollis* (Mb), *Naegleria Gruberi* (Ng), *Leishmania major* (Lm), *Leishmania braziliensis* (Lb), *Leishmania infantum* (Li), *Trypanosoma brucei* (Tb), *Trypanosoma cruzi* (Tc), *Plasmodium falciparum* (Pf), *Toxoplasma gondii* (Tg), *Tetrahymena thermophila* (Tt), *Paramecium tetraaurelia* (Pt), *Giardia lamblia* (Gl), *Trichomonas vaginalis* (Tv), *Phytophtora infestans* (Pi), *Phytophtora sojae* (Ps), *Micromonas pusilla* (Mp), *Volvox carteri* (Vc)

References

1. Huson DH, Richter DC, Rausch C, Dezulian T, Franz M et al. (2007) Dendroscope: An interactive viewer for large phylogenetic trees. BMC Bioinformatics 8: 460.
