# Supplementary material for: Thousands of Rab GTPases for the Cell Biologist
Source: PLoS Comput Biol. 2011 Oct 13;7(10):e1002217. doi: 10.1371/journal.pcbi.1002217 (PMC3192815; doi:10.1371/journal.pcbi.1002217)
Supplement: Figure S5 — Tissue specificity in expression of mouse Rabs. We analysed microarray data from the Mouse Exon Atlas (GEO accession: GSE15998) generated on an Affymetrix Mouse Exon 1.0 ST Array (GEO accession: GPL6193) downloaded from [121]. We mapped probes to genes using the R package provided in [122]. The data analysis has been performed in R using Bioconductor's ‘affy’ library. After applying RMA [123], we combined biological replicates by averaging their expression value. To transform continuous expression (B) into a discrete present/absent pattern (A) we chose a threshold (5.0) that maximises the agreement with the PCR data from Figure 8 while achieving a balanced number of false positives (28) and false negatives (25). (PDF) [file pcbi.1002217.s006.pdf]

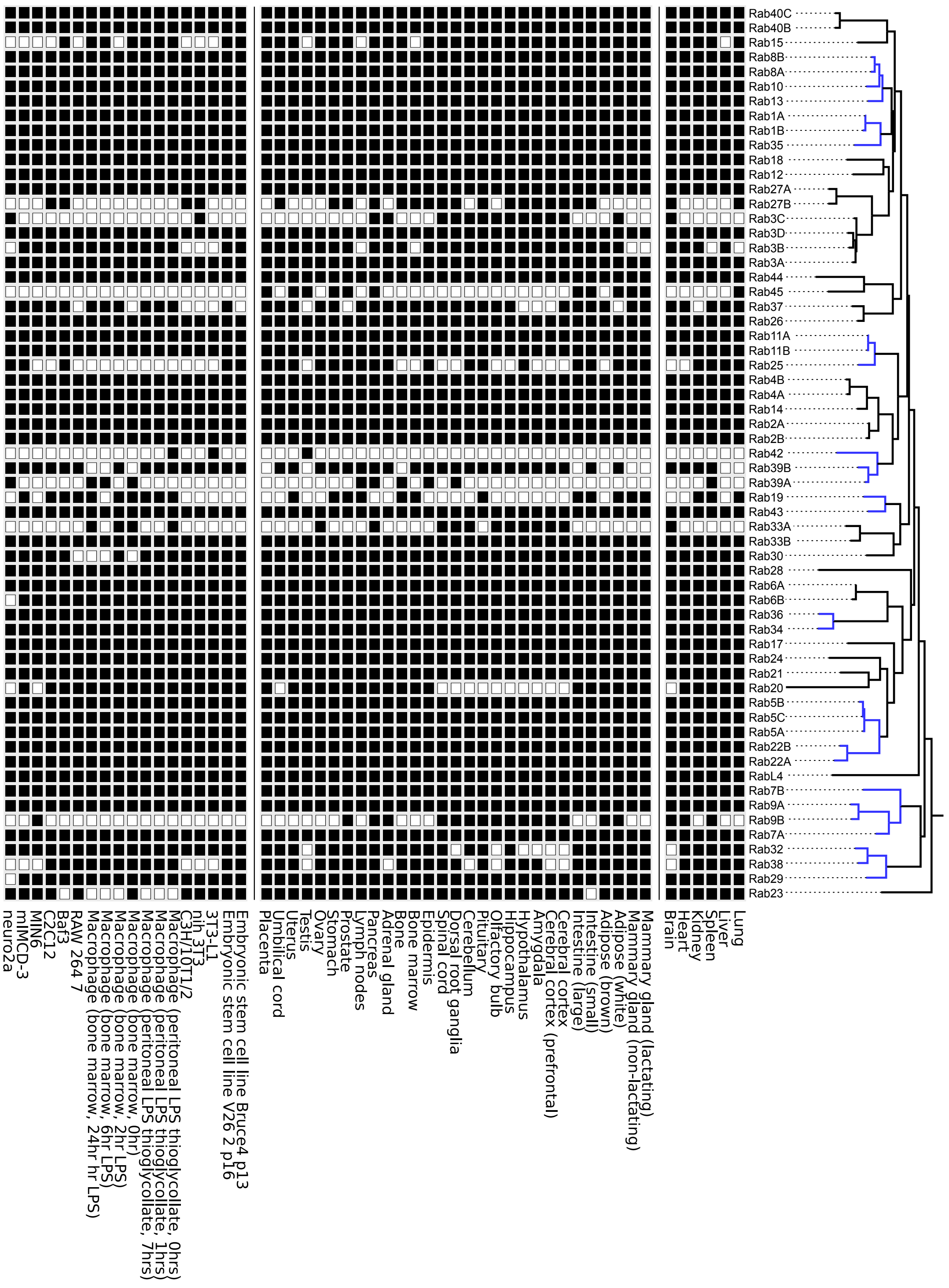

Figure S5. Tissue specificity in expression of mouse Rabs. We analysed microarray data from the Mouse Exon Atlas (GEO accession: GSE15998) generated on an Affymetrix Mouse Exon 1.0 ST Array (GEO accession: GPL6193) downloaded from [1]. We mapped probes to genes using the R package provided in [2]. The data analysis has been performed in R using Bioconductor's 'affy' library. After applying RMA [3], we combined biological replicates by averaging their expression value. To transform continuous expression

into a discrete present/absent pattern we chose a threshold (5.0) that maximises the agreement with the PCR data from **Figure 8** while achieving a balanced number of false positives (28) and false negatives (25).

References

1. Wu C, Orozco C, Boyer J, Leglise M, Goodale J et al. (2009) BioGPS: an extensible and customizable portal for querying and organizing gene annotation resources. Genome Biol 10: R130.
2. Risueno A, Fontanillo C, Dinger ME, De Las Rivas J (2010) GATExplorer: genomic and transcriptomic explorer; mapping expression probes to gene loci, transcripts, exons and ncRNAs. BMC Bioinformatics 11: 221.
3. Irizarry RA, Hobbs B, Collin F, Beazer-Barclay YD, Antonellis KJ et al. (2003) Exploration, normalization, and summaries of high density oligonucleotide array probe level data. Biostatistics 4: 249-264.
