# Supplementary material for: Thousands of Rab GTPases for the Cell Biologist
Source: PLoS Comput Biol. 2011 Oct 13;7(10):e1002217. doi: 10.1371/journal.pcbi.1002217 (PMC3192815; doi:10.1371/journal.pcbi.1002217)
Supplement: Figure S6 — Quantitative expression of Rabs in mouse tissues. Figure (A) plots the same data as shown in Figure S5 prior to the binarisation via thresholding. (B) shows the average expression across the mouse tissues (cell lines not included). (PDF) [file pcbi.1002217.s007.pdf]

(A)

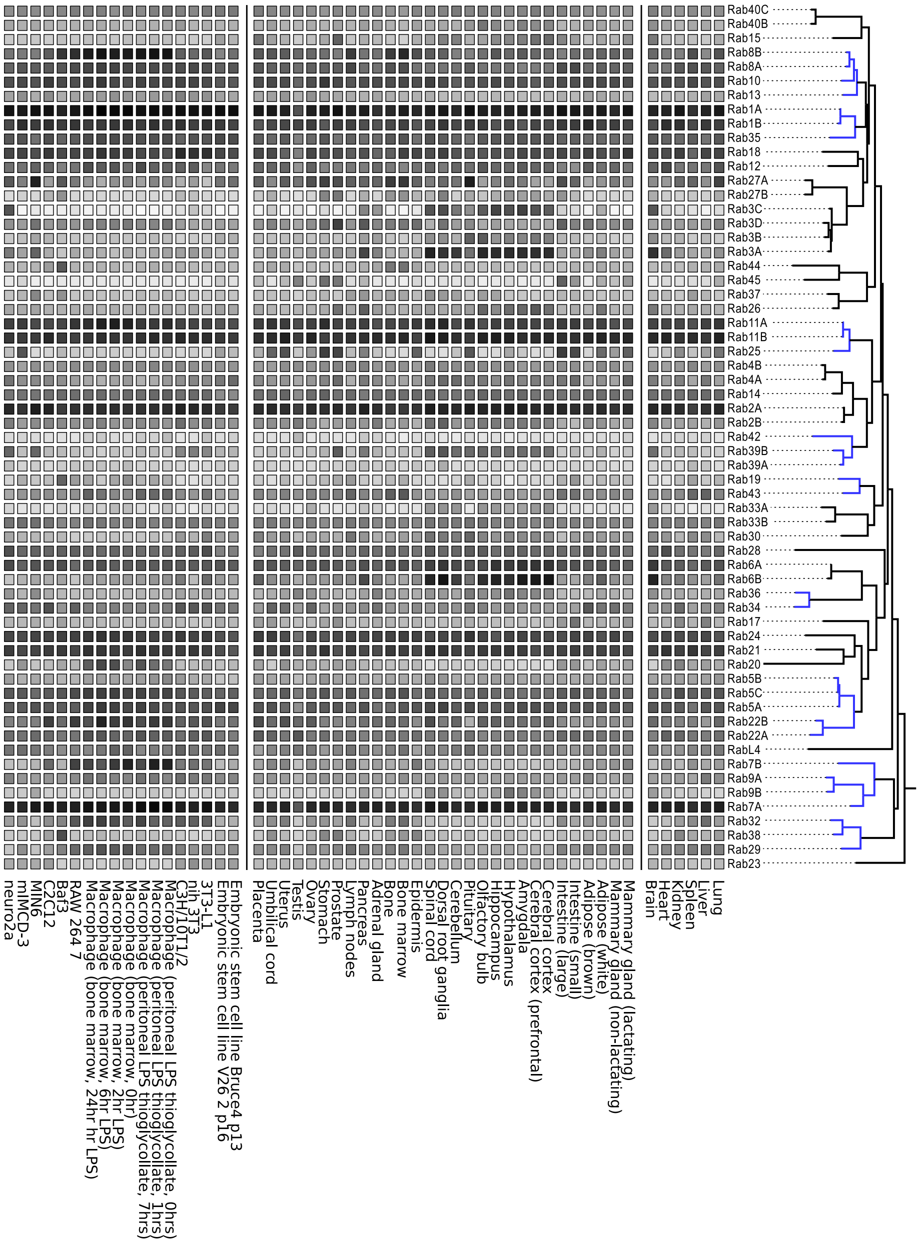
Rab40C
Rab40B
Rab15
Rab8B
Rab8A
Rab10
Rab13
Rab1A
Rab1B
Rab35
Rab18
Rab12
Rab27A
Rab27B
Rab3C
Rab3D
Rab3B
Rab3A
Rab44
Rab45
Rab37
Rab26
Rab11A
Rab11B
Rab25
Rab4B
Rab4A
Rab14
Rab2A
Rab2B
Rab42
Rab39B
Rab39A
Rab19
Rab43
Rab33A
Rab33B
Rab30
Rab28
Rab6A
Rab6B
Rab36
Rab34
Rab17
Rab24
Rab21
Rab20
Rab5B
Rab5C
Rab5A
Rab22B
Rab22A
RabL4
Rab7B
Rab9A
Rab9B
Rab7A
Rab32
Rab38
Rab29
Rab23
neuro2a
mIMCD-3
MIN6
C2C12
Baf3
RAW 264 7
Macrophage (bone marrow, 24hr hr LPS)
Macrophage (bone marrow, 6hr LPS)
Macrophage (bone marrow, 2hr LPS)
Macrophage (bone marrow, 0hr)
Macrophage (peritoneal LPS thioglycollate, 7hrs)
Macrophage (peritoneal LPS thioglycollate, 1hrs)
Macrophage (peritoneal LPS thioglycollate, 0hrs)
C3H/10T1/2
nih 3T3
3T3-L1
Embryonic stem cell line V26 2 p16
Embryonic stem cell line Bruce4 p13
Placenta
Umbilical cord
Uterus
Testis
Ovary
Stomach
Prostate
Lymph nodes
Pancreas
Adrenal gland
Bone
Bone marrow
Epidermis
Spinal cord
Dorsal root ganglia
Cerebellum
Pituitary
Olfactory bulb
Hippocampus
Hypothalamus
Amygdala
Cerebral cortex
Cerebral cortex (prefrontal)
Intestine (large)
Intestine (small)
Adipose (brown)
Adipose (white)
Mammary gland (non-lactating)
Mammary gland (lactating)
Brain
Heart
Spleen
Kidney
Liver
Lung

(B)

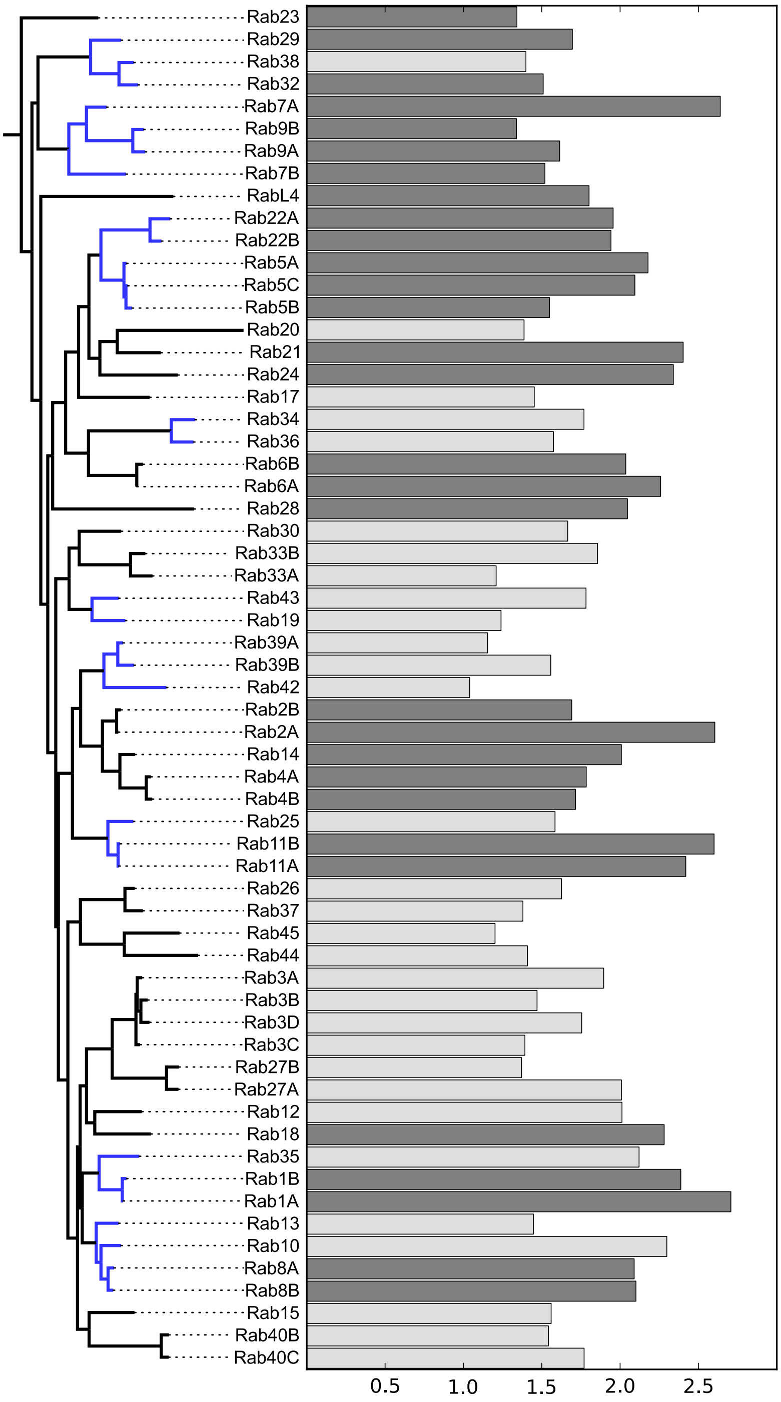

Figure S6. Quantitative expression of Rabs in mouse tissues. Figure (**A**) plots the same data as shown in **Figure S5** prior to the binarisation via thresholding. (**B**) shows the average expression across the mouse tissues (cell lines not included).
