# Supplementary material for: Thousands of Rab GTPases for the Cell Biologist
Source: PLoS Comput Biol. 2011 Oct 13;7(10):e1002217. doi: 10.1371/journal.pcbi.1002217 (PMC3192815; doi:10.1371/journal.pcbi.1002217)
Supplement: Figure S7 — Distribution of Rabs belonging to non-human subfamilies. The histogram details for each taxon how we classified those Rabs not belonging to human subfamilies. Subfamilies falling into the orange category have been previously described in the literature, whereas all other subfamilies result from clustering of the sequences as described in Materials and Methods . See Figure 4 for an overview of the number of subfamilies in each category. (PDF) [file pcbi.1002217.s008.pdf]

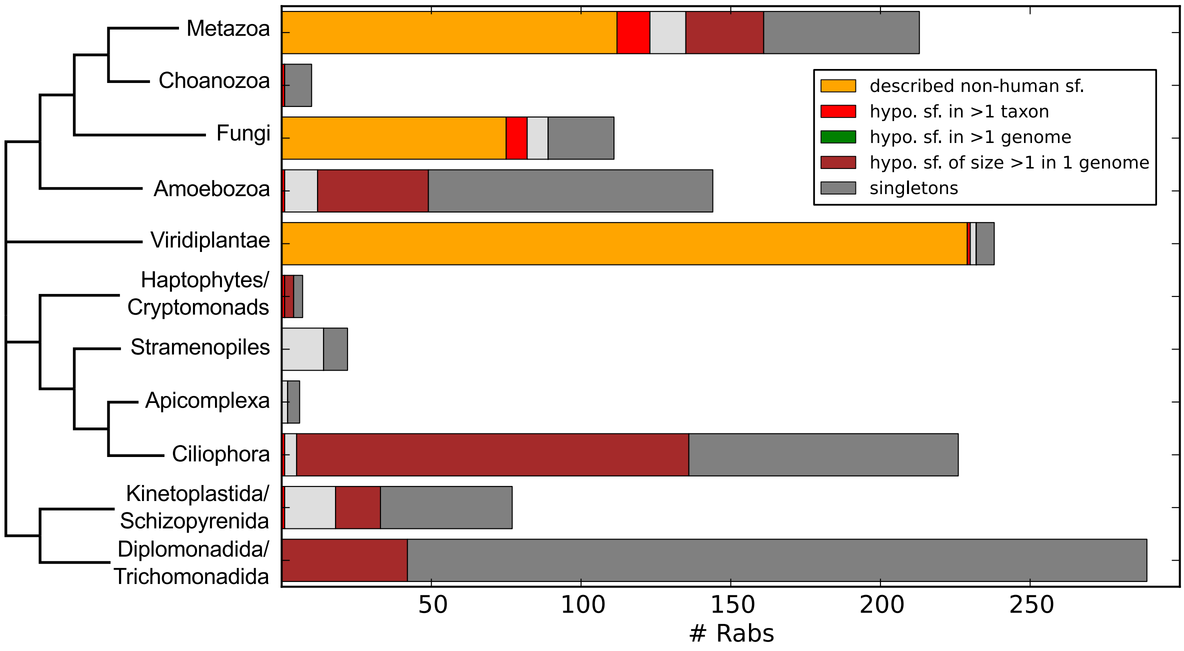

Figure S7. Distribution of Rabs belonging to non-human subfamilies. The histogram details for each taxon how we classified those Rabs not belonging to subfamilies also found in humans. Subfamilies falling into the orange category have been previously described in the literature, whereas all other subfamilies result from clustering of the sequences as described in **Materials and Methods**. See **Figure 4** for an overview of the number of subfamilies in each category.
