# Supplementary material for: Thousands of Rab GTPases for the Cell Biologist
Source: PLoS Comput Biol. 2011 Oct 13;7(10):e1002217. doi: 10.1371/journal.pcbi.1002217 (PMC3192815; doi:10.1371/journal.pcbi.1002217)
Supplement: Table S1 — Results of Rabifier validation. We list the accessions of the proteins used to perform the validations in Figure 2 . All Superfamily accessions refer to the release as of September 2009. As indicated in the upper table, four sequences from [32] have invalid IDs and are replaced by sequences with the same annotation from the newest release of the Trypansoma genome project (ftp://ftp.sanger.ac.uk/pub/databases/T.brucei_sequences/T.brucei_genome_v4/). Sequences missing either type of accession are those unique either to the respective paper or our automated scan of the full genome using the Rabifier. For the latter, we manually checked the sequences for being false positives, however, as we could not recover the full genomes used in the initial studies and the protein sequences predicted from fully sequenced genomes are not stable we expected certain amounts of discrepancies. In the last part of the table containing the results of the alternative strategy for M. brevicollis, ‘NOT A RAB’ stands for total lack of information allowing to infer that the protein may be a Rab, ‘RAB’ simply stands for lack of any subfamily annotation. The regular expression used to automatically scan the ‘region’ annotation for family- and subfamily-information was ‘(?:∧|\s)([\w]{2}rab{1}\w?\d{1,2}\w?)|(rab{1}\w?\d{1,2}\w?)|(rab{1})’. (PDF) [file pcbi.1002217.s009.pdf]

Table S1. Results of Rabifier validation.

	sequence accessions (Superfamily / paper)			reference classification	classification Rabifier
T. brucei [1]					
	Tb09.211.2330	re-places	TRYPtp3p8-g10.p1c_1 (invalid ID)	TbRab7	Rab7
	Tb09.244.2070	re-places	tryp_X_467d02.p1c (invalid ID)	TbRab1B	AtRabD1
	Tb10.389.0550	≈99%	AAC46991.1	TbRab5A	Rab5
	Tb10.6k15.1990	≈97%	AY370775.1/AAR14148.1	TbRab23	Rab23
	Tb10.70.6420	re-places	CONTIG11942 (invalid ID)	TbRab21	Rab21
	Tb11.01.5320		AAC46990.1	TbRab4	Rab4
FN	Tb11.01.6670	≈96%	AAG39035.1	TbRabX3	NOT A RAB
	Tb11.01.6890	≈99%	AY370774.1/AAR14147.1	TbRab2	Rab2
	Tb11.02.2160	≈97%	AAC78731.1	TbRab5B	Rab5
	Tb927.2.2130/ 25N14.200		AAQ15670.1	TbRab6	Rab6
	Tb927.6.3040/ Tb06.5F5.770	re-places	CONTIG12099 (invalid ID)	TbRab28	Rab28
	Tb927.8.4330/ Tb08.29H22.590		AAG39034.1	TbRab11	Rab11
	Tb927.8.4610/ Tb08.4A8.220		CAA68211.1	TbRabX1	KinetoSchizoRab X1
	Tb927.8.4620/ Tb08.4A8.230		CAA68210.1	TbRabX2	uzRabX3
	Tb927.8.4770/ Tb08.4A8.450		AAF37004.1	TbRab18	Rab18
	Tb927.8.890/ Tb08.12O16.530		AY370773.1/AAR14146.1	TbRab1A	Rab1
	Tb927.4.4220/ Tb04.1D20.300		—	—	uzRabX1
	Tb927.8.8140/ Tb08.28A12.160		—	—	uzRabX1
E. histolytica [2]					
	1.m00684		BAD82853.1	EhRabX5	RabX
	101.m00134	≈99%	BAB40671.1	EhRabD1	Rab1

	sequence accessions (Superfamily / paper)			reference classification	classification Rabifier
	102.m00088		BAD82827.1	EhRabC7	AtRabA1
	103.m00156		BAD82847.1	EhRabM1	enRabX10
	103.m00161	*100% identical to 103.m00156*			
	104.m00162	*99% identical to 323.m00049*			
	105.m00118	*93% identical to 39.m00244*			
	105.m00137		BAB40678.1	EhRab11	Rab11
	108.m00111		BAD82849.1	EhRabN1	enRabX5
	11.m00316		BAD82865.1	EhRabX17	RabX
	112.m00114		BAD82822.1	EhRab11	Rab11
FN	114.m00128		BAD82854.1	EhRabX6	NOT A RAB
	114.m00149		BAD82868.1	EhRabX20	Rab8
	12.m00305		BAD34974.1	EhRab7	Rab7
	121.m00115		BAD82823.1	EhRabC3	Rab35
	122.m00143		BAD34970.1	EhRab7	Rab7
	123.m00112		BAD82821.1	EhRab8	Rab8
	124.m00130		BAD82841.1	EhRabK1	enRabX9
	125.m00102	≈96%	AAB86482.1	EhRabA	enRabX3
	126.m00116		BAD82848.1	EhRabM2	enRabX10
	13.m00301		BAB40670.1	EhRabC1	Rab1
FN	14.m00335		BAD82877.1	EhRabX29	NOT A RAB
	140.m00075		BAB40672.1	EhRab21	Rab21
	141.m00085		BAD34971.1	EhRab7	Rab7
	141.m00095		BAD82878.1	EhRabX30	enRabX2
	144.m00086		BAD82879.1	EhRabX31	enRabX13
	145.m00080		BAD82842.1	EhRabK3	enRabX5
	146.m00105		BAB40675.1	EhRabF1	Rab5
	148.m00086		BAD82851.1	EhRabP1	enRabX8
	150.m00119		BAD82846.1	EhRabM3	enRabX10
	151.m00108		BAD82840.1	EhRabX4	RabX
	16.m00325	*100% identical to 141.m00095*			

	sequence accessions (Superfamily / paper)			reference classification	classification Rabifier
	161.m00089		BAD82832.1	EhRabF2	AtRabA5
	162.m00073		BAD82856.1	EhRabX8	enRabX4
	170.m00128		BAB40669.1	EhRab1	Rab1
	177.m00099		BAD82845.1	EhRabL1	enRabX7
	177.m00124		BAD34972.1	EhRab7	Rab7
	18.m00284		BAD82852.1	EhRabP2	enRabX8
	18.m00336		BAB40676.1	EhRabF5	Rab22
	183.m00117		BAD82880.1	EhRabX32	Rab1
	186.m00126		BAD82828.1	EhRabC8	Rab18
	198.m00117	≈100%	BAD82875.1	EhRabX27	RabX
	199.m00093		BAD82834.1	EhRabX1	Rab11
	2.m00606		BAD82874.1	EhRabX26	Rab5
	20.m00304		BAB40680.1	EhRab11	Rab1
	20.m00332		BAD82859.1	EhRabX11	enRabX7
	201.m00118		BAD82869.1	EhRabX21	RabX
	205.m00097		BAD82871.1	EhRabX23	Rab6
	21.m00285		*fragment of 31.m00204*		
	22.m00318		BAD82881.1	EhRabX33	enRabX11
	226.m00092		BAD34973.1	EhRab7	Rab7
	227.m00074		BAD82882.1	EhRabX34	Rab18
	241.m00091		BAD82876.1	EhRabX28	enRabX1
	250.m00079		BAD82838.1	EhRabX2	Rab7
	260.m00082		BAD82835.1	EhRab2	Rab2
	261.m00062		*100% identical to 162.m00073*		
	27.m00248		BAD82864.1	EhRabX16	AtRabA5
	285.m00064	≈99%	BAB40679.1	EhRab11	Rab11
	30.m00257		BAB40673.1	EhRab5	Rab5
	304.m00066		BAD82850.1	EhRabN2	enRabX5
	313.m00055		BAD82843.1	EhRabK2	enRabX9
	316.m00031		BAD82836.1	EhRab2	Rab2

	sequence accessions (Superfamily / paper)			reference classification	classification Rabifier
	319.m00059		BAD82820.1	EhRab7	Rab7
	323.m00049		BAD82866.1	EhRabX18	enRabX6
	323.m00050		BAD82863.1	EhRabX15	Rab1
	333.m00048		BAB40674.1	EhRab7	Rab7
	337.m00051		BAD82883.1	EhRabX35	RabX
	34.m00229		BAD82831.1	EhRabF3	Rab11
	34.m00273		BAD82829.1	EhRabD2	Rab1
	350.m00049		BAD82819.1	EhRab1	Rab1
	36.m00198		BAD82870.1	EhRabX22	Rab6
	364.m00047	*100% identical to 194.m00122*			
	37.m00226	*99% identical to 36.m00198*			
	37.m00253		BAD82839.1	EhRabX3	RabX
	372.m00053	*100% identical to 144.m00086*			
	39.m00244	≈98%	BAD82867.1	EhRabX19	enRabX12
	4.m00593		BAD82862.1	EhRabX14	Rab8
	45.m00174	*98% identical to 5.m00400*			
	46.m00241		BAD82826.1	EhRabC6	RabI
	490.m00038		BAD82884.1	EhRabK4	enRabX9
	491.m00024	*99% identical to 22.m00318*			
	5.m00400		BAB40677.1	EhRabH	enRabX3
FN	5.m00440		BAD82885.1	EhRabX36	enRabX1
	59.m00166		BAD82830.1	EhRabF4	AtRabA1
	59.m00179		BAD82825.1	EhRabC5	Rab1
	59.m00183		BAD82833.1	EhRabI2	RabX
	6.m00402		BAD82824.1	EhRabC4	Rab1
	6.m00410		BAD82858.1	EhRabX10	RabX
	60.m00181		BAD82857.1	EhRabX9	RabX
FN	62.m00151		BAD82855.1	EhRabX7	NOT A RAB
	7.m00414		BAD82872.1	EhRabX24	Rab1

	sequence accessions (Superfamily / paper)		reference classification	classification Rabifier
	7.m00469	BAD34969.1	EhRab7	Rab7
	703.m00016	*100% identical to 241.m00091*		
	725.m00007	BAD34976.1	EhRab11	Rab11
	76.m00156	BAD82861.1	EhRabX13	Rab8
	78.m00171	BAD34977.1	EhRabC2	Rab1
	796.m00014	*100% identical to 241.m00091*		
	8.m00400	BAD82837.1	EhRab2	Rab2
	83.m00197	BAD34975.1	EhRab7	Rab7
	95.m00136	BAD82873.1	EhRabX25	RabX
	95.m00153 ≈100%	AAK62316.1	EhRab8	Rab8
	97.m00134	AAF37308.1	EhRabB	DmRabX
	—	BAD82860.1	EhRabX12	Rab1
	144.m00089	—	—	RabX
	194.m00122	—	—	RabX
	31.m00204	—	—	enRabX9
	349.m00044	—	—	AtRabA4
Monosiga brevicollis (Rabifier)				
	jgi|Monbr1|12170|fgenesh1_pg.scaffold_35000091		MbRabX5	RabX
	jgi|Monbr1|13239|fgenesh1_pg.scaffold_421000001		MbRab21	Rab21
	jgi|Monbr1|14340|e_gw1.3.572.1		MbRabL4	RabL4
	jgi|Monbr1|15030|e_gw1.4.429.1		MbRabX1	Rab6
	jgi|Monbr1|15717|e_gw1.5.339.1		MbRab7	Rab7
	jgi|Monbr1|17807|estExt_gwp_gw1.C_30598		MbRab32A	Rab32
	jgi|Monbr1|18358|estExt_gwp_gw1.C_50375		MbRab4	Rab4
	jgi|Monbr1|21678|fgenesh2_pm.scaffold_26000005		MbRab14	Rab14
	jgi|Monbr1|25453|fgenesh2_pg.scaffold_10000103		MbRabX3	Rab31
	jgi|Monbr1|25870|fgenesh2_pg.scaffold_12000019		MbRab23	Rab23
	jgi|Monbr1|26044|fgenesh2_pg.scaffold_12000193		MbRabX2	Rab1
	jgi|Monbr1|27837|fgenesh2_pg.scaffold_22000022		MbRab28	Rab28
	jgi|Monbr1|32315|estExt_fgenesh2_pg.C_90149		MbRabX4B	Rab8

	sequence accessions (Superfamily / paper)	reference classification	classification Rabifier
	jgi|Monbr1|32662|estExt_fgenesh2_pg.C_120120	MbRab6	Rab6
	jgi|Monbr1|34573|estExt_fgenesh2_pg.C_410015	MbRab8	Rab8
	jgi|Monbr1|34712|estExt_fgenesh2_pg.C_470011	MbRab5	Rab5
	jgi|Monbr1|35113|estExt_fgenesh2_pm.C_70039	MbRab18	Rab18
	jgi|Monbr1|35292|estExt_fgenesh2_pm.C_310004	MbRab9	Rab9
	jgi|Monbr1|35328|estExt_fgenesh2_pm.C_410003	MbRab11	Rab11
	jgi|Monbr1|35367|estExt_fgenesh2_kg.C_200001	MbRabX4A	Rab1
	jgi|Monbr1|36068|estExt_fgenesh1_pg.C_40009	MbRab22	Rab22
	jgi|Monbr1|37450|estExt_fgenesh1_pg.C_130154	MbRab1	Rab1
	jgi|Monbr1|38811|estExt_fgenesh1_pg.C_320017	MbRab2	Rab2
	jgi|Monbr1|38928|estExt_fgenesh1_pg.C_340049	MbRab32B	Rab32
	jgi|Monbr1|8415|fgenesh1_pg.scaffold_11000039	MbRab29	Rab29
Monosiga brevicollis (Alt. strategy)			
	jgi|Monbr1|12170|fgenesh1_pg.scaffold_35000091	MbRabX5	RAB
	jgi|Monbr1|13239|fgenesh1_pg.scaffold_421000001	MbRab21	NOT A RAB
	jgi|Monbr1|14340|e_gw1.3.572.1	MbRabL4	NOT A RAB
	jgi|Monbr1|15030|e_gw1.4.429.1	MbRabX1	RAB
	jgi|Monbr1|15717|e_gw1.5.339.1	MbRab7	NOT A RAB
	jgi|Monbr1|17807|estExt_gwp_gw1.C_30598	MbRab32A	Rab38
	jgi|Monbr1|18358|estExt_gwp_gw1.C_50375	MbRab4	Rab4
	jgi|Monbr1|21678|fgenesh2_pm.scaffold_26000005	MbRab14	NOT A RAB
	jgi|Monbr1|25453|fgenesh2_pg.scaffold_10000103	MbRabX3	Rab5
	jgi|Monbr1|25870|fgenesh2_pg.scaffold_12000019	MbRab23	NOT A RAB
	jgi|Monbr1|26044|fgenesh2_pg.scaffold_12000193	MbRabX2	RAB
	jgi|Monbr1|27837|fgenesh2_pg.scaffold_22000022	MbRab28	NOT A RAB
	jgi|Monbr1|32315|estExt_fgenesh2_pg.C_90149	MbRabX4B	RAB
	jgi|Monbr1|32662|estExt_fgenesh2_pg.C_120120	MbRab6	Rab6
	jgi|Monbr1|34573|estExt_fgenesh2_pg.C_410015	MbRab8	Rab8
	jgi|Monbr1|34712|estExt_fgenesh2_pg.C_470011	MbRab5	Rab5
	jgi|Monbr1|35113|estExt_fgenesh2_pm.C_70039	MbRab18	Rab18

	sequence accessions (Superfamily / paper)	reference classification	classification Rabifier
	jgi|Monbr1|35292|estExt_fgenesh2_pm.C_310004	MbRab9	NOT A RAB
	jgi|Monbr1|35328|estExt_fgenesh2_pm.C_410003	MbRab11	Rab11
	jgi|Monbr1|35367|estExt_fgenesh2_kg.C_200001	MbRabX4A	RAB
	jgi|Monbr1|36068|estExt_fgenesh1_pg.C_40009	MbRab22	Rab5
	jgi|Monbr1|37450|estExt_fgenesh1_pg.C_130154	MbRab1	Rab1
	jgi|Monbr1|38811|estExt_fgenesh1_pg.C_320017	MbRab2	Rab2
	jgi|Monbr1|38928|estExt_fgenesh1_pg.C_340049	MbRab32B	Rab38
	jgi|Monbr1|8415|fgenesh1_pg.scaffold_11000039	MbRab29	NOT A RAB

We list the accessions of the proteins used to perform the validations in **Figure 2**. All Superfamily accessions refer to the release as of September 2009. As indicated in the upper table, four sequences from [1] have invalid IDs and are replaced by sequences with the same annotation from the newest release of the Trypansoma genome project (ftp://ftp.sanger.ac.uk/pub/databases/T.brucei_sequences/T.brucei_genome_v4/). Sequences missing either type of accession are those unique either to the respective paper or our automated scan of the full genome using the Rabifier. For the latter, we manually checked the sequences for being false positives, however, as we could not recover the full genomes used in the initial studies and the protein sequences predicted from fully sequenced genomes are not stable we expected certain amounts of discrepancies.

In the last part of the table containing the results of the alternative strategy for *M. brevicollis*, ‘NOT A RAB’ stands for total lack of information allowing to infer that the protein may be a Rab, ‘RAB’ simply stands for lack of any subfamily annotation. The regular expression used to automatically scan the ‘region’ annotation for family- and subfamily-information was '(?:^|\s)([\w]{2} rab{1}\w?\d{1,2}\w?)|(rab{1}\w?\d{1,2}\w?)|(rab{1})'.

References

1. Ackers JP, Dhir V, Field MC (2005) A bioinformatic analysis of the RAB genes of Trypanosoma brucei. Mol Biochem Parasitol 141: 89-97.
2. Saito-Nakano Y, Loftus BJ, Hall N, Nozaki T (2005) The diversity of Rab GTPases in Entamoeba histolytica. Exp Parasitol 110: 244-252.
