# Supplementary material for: Thousands of Rab GTPases for the Cell Biologist
Source: PLoS Comput Biol. 2011 Oct 13;7(10):e1002217. doi: 10.1371/journal.pcbi.1002217 (PMC3192815; doi:10.1371/journal.pcbi.1002217)
Supplement: Table S2 — Accessions of all sequences. Uniprot [124] accessions of all sequences used to generate the phylogenetic trees in Figures 4 – 7 and Figures S3, S4. The Uniprot human Rab42 sequence is most probably only a fragment, hence in all cases the alternative sequence from Superfamily [41] is used. In case of multidomain proteins (human and mouse Rab44 and Rab45), alignments were generated only using the designated residues. Isoforms of the same Rab subfamily are distinguished by prime symbols. Abbreviations: Homo sapiens (Hs), Mus musculus (Mm), Monosiga brevicollis (Mb), Naegleria Gruberi (Ng), Leishmania major (Lm), Leishmania braziliensis (Lb), Leishmania infantum (Li), Trypanosoma brucei (Tb), Trypanosoma cruzi (Tc), Plasmodium falciparum (Pf), Toxoplasma gondii (Tg), Tetrahymena thermophila (Tt), Paramecium tetraaurelia (Pt), Giardia lamblia (Gl), Trichomonas vaginalis (Tv), Phytophtora infestans (Pi), Phytophtora sojae (Ps), Micromonas pusilla (Mp), Volvox carteri (Vc). (PDF) [file pcbi.1002217.s010.pdf]

Table S2. Accessions of all sequences.

Species	GTPase	Uniprot	Superfamily	which residues
		RABs		
Hs	Rab1A	P62820		
Hs	Rab1B	Q9H0U4		
Hs	Rab2A	P61019		
Hs	Rab2B	Q8WUD1		
Hs	Rab3A	P20336		
Hs	Rab3B	P20337		
Hs	Rab3C	Q96E17		
Hs	Rab3D	O95716		
Hs	Rab4A	P20338		
Hs	Rab4B	P61018		
Hs	Rab5A	P20339		
Hs	Rab5B	P61020		
Hs	Rab5C	P51148		
Hs	Rab6A	P20340		
Hs	Rab6B	Q9NRW1		
Hs	Rab6C	Q9H0N0		
Hs	Rab7A	P51149		
Hs	Rab7B	Q96AH8		
Hs	Rab8A	P61006		
Hs	Rab8B	Q92930		
Hs	Rab9A	P51151		
Hs	Rab9B	Q9NP90		
Hs	Rab10	P61026		
Hs	Rab11A	P62491		
Hs	Rab11B	Q15907		
Hs	Rab12	Q6IQ22		
Hs	Rab13	P51153		

Species	GTPase	Uniprot	Superfamily	which residues
Hs	Rab14	P61106		
Hs	Rab15	P59190		
Hs	Rab17	Q9H0T7		
Hs	Rab18	Q9NP72		
Hs	Rab19	A4D1S5		
Hs	Rab20	Q9NX57		
Hs	Rab21	Q9UL25		
Hs	Rab22A	Q9UL26		
Hs	Rab22B (=Rab31)	Q13636		
Hs	Rab23	Q9ULC3		
Hs	Rab24	Q969Q5		
Hs	Rab25	P57735		
Hs	Rab26	Q9ULW5		
Hs	Rab27A	P51159		
Hs	Rab27B	O00194		
Hs	Rab28	P51157		
Hs	Rab29 (=Rab7L1)	O14966		
Hs	Rab30	Q15771		
Hs	Rab32	Q13637		
Hs	Rab33A	Q14088		
Hs	Rab33B	Q9H082		
Hs	Rab34	Q9BZG1		
Hs	Rab35	Q15286		
Hs	Rab36	O95755		
Hs	Rab37	Q96AX2		
Hs	Rab38	P57729		
Hs	Rab39A	Q14964		
Hs	Rab39B	Q96DA2		
Hs	Rab40A	Q8WXH6		

**Species**	**GTPase**	**Uniprot**	**Superfamily**	**which residues**
Hs	Rab40B	Q12829		
Hs	Rab40C	Q96S21		
Hs	Rab41	Q5JT25		
Hs	Rab42	Q8N4Z0	ENSP00000373941	
Hs	Rab43	Q86YS6		
Hs	Rab44	Q7Z6P3		514 to end
Hs	Rab45/Rasef	Q8IZ41		512 to end
Hs	RabL4	Q9BW83		
Mm	Rab1A	P62821		
Mm	Rab1B	Q9D1G1		
Mm	Rab2A	P53994		
Mm	Rab2B	P59279		
Mm	Rab3A	P63011		
Mm	Rab3B	Q9CZT8		
Mm	Rab3C	P62823		
Mm	Rab3D	P35276		
Mm	Rab4A	P56371		
Mm	Rab4B	Q91ZR1		
Mm	Rab5A	Q9CQD1		
Mm	Rab5B	P61021		
Mm	Rab5C	P35278		
Mm	Rab6A	P35279		
Mm	Rab6B	P61294		
Mm	Rab7A	P51150		
Mm	Rab7B	Q8VEA8		
Mm	Rab8A	P55258		
Mm	Rab8B	P61028		
Mm	Rab9A	Q9R0M6		
Mm	Rab9B	Q8BHH2		
Mm	Rab10	P61027		

Species	GTPase	Uniprot	Superfamily	which residues
Mm	Rab11A	P62492		
Mm	Rab11B	P46638		
Mm	Rab12	P35283		
Mm	Rab13	Q9DD03		
Mm	Rab14	Q91V41		
Mm	Rab15	Q8K386		
Mm	Rab17	P35292		
Mm	Rab18	P35293		
Mm	Rab19	P35294		
Mm	Rab20	P35295		
Mm	Rab21	P35282		
Mm	Rab22A	P35285		
Mm	Rab22B	Q921E2		
Mm	Rab23	P35288		
Mm	Rab24	P35290		
Mm	Rab25	Q9WTL2		
Mm	Rab26	Q504M8		
Mm	Rab27A	Q9ERI2		
Mm	Rab27B	Q99P58		
Mm	Rab28	Q99KL7		
Mm	Rab29	Q91YQ1		
Mm	Rab30	Q923S9		
Mm	Rab32	Q9CZE3		
Mm	Rab33A	P97950		
Mm	Rab33B	O35963		
Mm	Rab34	Q64008		
Mm	Rab35	Q6PHN9		
Mm	Rab36	Q8CAM5		
Mm	Rab37	Q9JKM7		
Mm	Rab38	Q8QZZ8		

Species	GTPase	Uniprot	Superfamily	which residues
Mm	Rab39A	Q8BHD0		
Mm	Rab39B	Q8BHC1		
Mm	Rab40B	Q8VHP8		
Mm	Rab40C	Q8VHQ4		
Mm	Rab42	Q0PD08		
Mm	Rab43	Q8CG50		
Mm	Rab44	Q8CB87		518 to end
Mm	Rab45	Q5RI75		400 to end
Mm	RabL4	Q9D0P8		
Ng	Rab14	D2VWL1		
Lm	Rab14	Q4QIN6		
Lb	Rab14	A4H506		
Li	Rab14	A4HT72		
Tc	Rab14	Q4DTS0		
Tc	Rab14'	Q4DT54		
Ng	Rab4	D2VQV4		
Lm	Rab4	Q4Q5N4		
Lb	Rab4	A4HK33		
Li	Rab4	A4I7N3		
Tb	Rab4	Q382G2		
Tc	Rab4	Q4CZF2		
Ng	Rab2	D2V8T4		
Ng	Rab2'	D2VS55		
Lm	Rab2	Q4Q573		
Lb	Rab2	A4HKJ3		
Li	Rab2	A4I825		
Tb	Rab2	Q381R1		
Tc	Rab2'	Q4D5S8		
Mb	Rab2	A9VAA2		
Dd	Rab2	P36409		

Species	GTPase	Uniprot	Superfamily	which residues
Dd	Rab2'	Q559X6		
Mb	Rab4	A9UUH9		
Dd	Rab4	Q54DA7		
Mb	Rab14	A9V8B6		
Dd	Rab14	P36410		
Pf	Rab2	Q8I5A9		
Tg	Rab4	B6KAY6		
Tg	Rab2	B6K8Q3		
Tt	Rab2	Q24GI2		
Tt	Rab2'	Q247T2		
Tt	Rab2''	E1CAY7		
Tt	Rab4	Q22SM7		
Tt	Rab4'	Q22ZG1		
Pt	Rab2	A0CW65		
Pt	Rab2'	Q3SDL7		
Pt	Rab2''	Q3SD50		
Ng	Rab32		jgi|Naegr1|4014|gw1.1.472.1	
Ng	Rab32'		jgi|Naegr1|56124| estExt_fgeneshHS_kg.C_170018	
Ng	Rab32''		jgi|Naegr1|60792| fgeneshNG_pm.scaffold_400000<br>07	
Ps	Rab23		jgi|Physo1_1|131661| estExt_fgenesh1_pg.C_170003	
Ps	Rab32		jgi|Physo1_1|122917| estExt_Genewise1.C_1170006	
Ps	Rab7		jgi|Physo1_1|108727| estExt_fgenesh1_pm.C_270012	
Gl	Rab32	A8B7Z5		
Tv	Rab7	Q4G2D0		
Tv	Rab7'	Q4G2D2		
Tv	Rab7''	A2FJT8		
Tb	Rab23	Q38AB6		

Species	GTPase	Uniprot	Superfamily	which residues
Lm	Rab23	Q4Q0D5		
Ng	Rab23	D2V6I8		
Ng	Rab7	D2VR26		
Ng	Rab7'	D2VAL1		
Tb	Rab7	Q38DS4		
Lm	Rab7	Q9N2P5		
Tg	Rab23	B6KMN9		
Tg	Rab7	B6KHB1		
Pf	Rab7	Q9NFG0		
Pi	Rab23	D0NED5		
Pi	Rab32	D0MQZ4		
Pi	Rab7	D0MZH3		
Mb	Rab7	A9UUX0		
Dd	Rab7	P36411		
Dd	Rab7'	Q54K69		
Mb	Rab32	A9URF9		
Mb	Rab32'	A9VB09		
Dd	Rab32	Q54QR3		
Dd	Rab32'	Q55E31		
Dd	Rab32''	Q8MXQ2		
Ps	Rab28		jgi|Physo1_1|158859| C_scaffold_162000002	
Ps	RabL4		jgi|Physo1_1|133189| estExt_fgenesh1_pg.C_240158	
Ng	Rab28		jgi|Naegr1|33099|e_gw1.20.88.1	
Lm	Rab28	Q4Q7C3		
Tb	Rab28	Q583N0		
Pi	Rab28	D0P3T6		
Pi	RabL4	D0P2B3		
Mp	RabL4	C1N2T5		
Vc	RabL4	D8TQE3		

Species	GTPase	Uniprot	Superfamily	which residues
Mp	Rab28	C1MLI7		
Mb	RabL4	A9URF3		
RABLs				
Hs	RabL2A	Q9UBK7		
Hs	RabL2B	Q9UNT1		
Hs	RabL3	Q5HYI8		
Hs	RabL5	Q9H7X7		
ARFs				
Hs	Arf1	P84077		
Hs	Arf3	P61204		
Hs	Arf4	P18085		
Hs	Arf5	P84085		
Hs	Arf6	P62330		
RAS'				
Hs	DIRas1	O95057		
Hs	DIRas2	Q96HU8		
Hs	DIRas3	O95661		
Hs	ERas	Q7Z444		
Hs	HRas	P01112		
Hs	KRas	P01116		
Hs	NRas	P01111		
Hs	RRas	P10301		
Hs	RRas2	P62070		
Hs	NKIRas1	Q9NYS0		
Hs	NKIRas2	Q9NYR9		
Mm	kRas	P3		
Mb	kRas	A9V9R9		
RHOs				
Hs	RhoA	P61586		
Hs	RhoB	P62745		

Species	GTPase	Uniprot	Superfamily	which residues
Hs	RhoC	P08134		
Hs	RhoD	O00212		
Hs	RhoE	P61587		
Hs	RhoF	Q9HBH0		
Hs	RhoG	P84095		
Hs	RhoH	Q15669		
Hs	RhoJ	Q9H4E5		
Hs	RhoQ	P17081		
Hs	RhoN	P52198		
Hs	RhoT1	Q8IXI2		
Hs	RhoT2	Q8IXI1		
Hs	RhoU	Q7L0Q8		
Hs	RhoV	Q96L33		
RANs				
Hs	Ran	P62826		
Hs	Ran_iso1	A8K3Z8		
Hs	Ran_iso2	B5MDF5		
Others				
Hs	Rem1	O75628		
Hs	Rem2	Q8IYK8		
Hs	RERG	Q96A58		
Hs	SRPRB	Q9Y5M8		

Uniprot [1] accessions of all sequences used to generate the phylogenetic trees in **Figures 5, 6, 8** and **Figures S3, S4**. The Uniprot human Rab42 sequence is most probably only a fragment, hence in all cases the alternative sequence from Superfamily [2] is used. In case of multidomain proteins (human and mouse Rab44 and Rab45), alignments were generated only using the designated residues. Isoforms of the same Rab subfamily are distinguished by prime symbols.

Abbreviations: *Homo sapiens* (Hs), *Mus musculus* (Mm), *Monosiga brevicollis* (Mb), *Naegleria gruberi* (Ng), *Leishmania major* (Lm), *Leishmania braziliensis* (Lb), *Leishmania infantum* (Li), *Trypanosoma brucei* (Tb), *Trypanosoma cruzi* (Tc), *Plasmodium falciparum* (Pf), *Toxoplasma gondii* (Tg), *Tetrahymena thermophila* (Tt), *Paramecium tetraaurelia* (Pt), *Giardia lamblia* (Gl), *Trichomonas vaginalis* (Tv), *Phytophtora infestans* (Pi), *Phytophtora sojae* (Ps), *Micromonas pusilla* (Mp), *Volvox carteri* (Vc)

References

1. The Uniprot Consortium (2010) The Universal Protein Resource (UniProt) in 2010. Nucleic Acids Res 38: D142-8.
2. Wilson D, Pethica R, Zhou Y, Talbot C, Vogel C et al. (2009) SUPERFAMILY—sophisticated comparative genomics, data mining, visualization and phylogeny. Nucleic Acids Res 37: D380-6.
