# Supplementary material for: Thousands of Rab GTPases for the Cell Biologist
Source: PLoS Comput Biol. 2011 Oct 13;7(10):e1002217. doi: 10.1371/journal.pcbi.1002217 (PMC3192815; doi:10.1371/journal.pcbi.1002217)
Supplement: Table S3 — Primer sequences used to characterise mouse Rabs. (PDF) [file pcbi.1002217.s011.pdf]

Table S3. Primer sequences to characterise mouse Rabs.

Rab	Forward primer	Tm/°C	Reverse primer	Tm/°C	PCR product size (bp)
1a	atgtgacagatcaggagtcc	57.3	TGACTGCTTGACTGGAGTGC	59.4	326
1b	actgaccaggagtcctacgc	61.4	TAGCAGCAGCCACCGCTAGC	63.5	335
2a	cgttcaaccacttgacaacc	57.3	TCAACAGCAGCCTCCCCCTG	63.5	359
2b	gacctcatggttagaggacg	59.4	TCAGAAACGTTCTGCTGAGG	57.3	333
3a	ttaatgcagtgcaggactgg	57.3	CAGGCACAATCCTGATGAGG	59.4	329
3b	cacagcctactaccgtgggg	63.5	TGGGGTGTCTGAGAGCCGCG	65.5	367
3c	accacagcctactatcgagg	59.4	TAGCAGCCACAGTTGGGCTG	61.4	345
3d	aaccaggagtccttcaccgc	61.4	CTAACAGCTGCAGCTGCTCG	61.4	329
4a	ggggcactccttgtctatga	61.4	GCACTGGTTTCCAGGAACAT	59.4	331
4b	gcttggctaactgatgcccg	61.4	CTGGCGGAGGGATATGTCGC	63.5	288
5a	accgaggagcacaagcagcc	63.5	CTACAACACTGGCTTCTGGC	59.4	371
5b	caggaaacgtttgctcgggc	61.4	AACACTGGCTCTTGTTCTGC	57.3	325
5c	acagatacatttgcacgggc	57.3	CTCTCCTGCAGATCCACACC	61.4	336
6a	attcgagactccactgtggc	59.4	CTTCATTGACTGGTTGCTCC	57.3	364
6b	gattgatgacgtcaggacgg	59.4	GCTCCTGAGGCTTGTCCAGC	63.5	275
7a	ccccaacactttcaaaaccc	57.3	TGGCCCGGTCATTCTTGTCC	61.4	320
7b	tccagagtcctttgaagccc	59.4	AGCAGCATCTGCTCTTTGGC	59.4	323
8a	agtcctttgacaacatccgg	57.3	ACTGCACCGGAAGAAGCTGG	61.4	332
8b	tgctggtctatgacatcacc	57.3	ACTGGTCTTCTTAGACCGGC	59.4	347
9a	gtgtcgatgattcacagagc	57.3	AAGATGAGTTTGGCTTGGGC	57.3	336
9b	ccggcagagctttgagaacc	61.4	GAAGAACTTGCTTTGGAGCC	57.3	324
10	gtgtatgacatcaccaacgg	57.3	GCAGCACTTGCTCTTCCAGC	61.4	339
11a	cgatggctgaaagaactgag	57.3	GACAGCACTGCACCTTTGGC	61.4	331
11b	aaggagctgcgggatcatgc	61.4	ACAGGCTCTGGCAGCACTGC	63.5	337
12	caagaccagcctgatggagc	61.4	TTCTTGGCCTGCTGTGTCCC	61.4	136
13	attaccgtggagccatgggc	61.4	TCAGGCCAGTGCTTGAGGGC	63.5	339
14	agctgcaggtgcgctcatgg	63.5	TGGGGTTCACTGGTTAGCCG	61.4	372
15	cagtgagcgctcctatcagc	61.4	GAGTTTGCTGGGCCCTCAGG	63.5	345
17	ggattcctttcacaaggccc	59.4	ATCGTGCACAGCACTGGCGC	63.5	335
18	ttatagaggtgcacagggag	57.3	CACAGAGCAGTAACCGCCGC	63.5	325
19	atgatctcacacggcggtcc	61.4	TCTCATTGGGGACCTGGGCC	63.5	284
20	caaagtggacctgacctcgg	61.4	CCGGATCTAGTCTGTTTGGG	59.4	349
21	taccgagattcgaacggagc	59.4	CCGCTGCTCTGAGCTTGAGG	63.5	278
22a	tgagagagctccgccagcac	63.5	AGCAGCTTCGCTTTGGCTCC	61.4	267

Rab	Forward primer	Tm/°C	Reverse primer	Tm/°C	PCR product size (bp)
22b	gctcctatgtactaccgagg	59.4	TTCCCAAGCTTGATCCCGCC	61.4	341
23	tataccaaccgcactggtgc	59.4	GTTTCTGGTTCTCTTGGTCC	57.3	349
24	acctcactgacagcagcagc	61.4	AGTAAGGGTTTGCCTTCTGG	57.3	325
25	ggcactcctggtatttgacc	59.4	GGATCCTGTCCAGCTTGGGC	63.5	342
26	tgctgctctacgacatcacc	59.4	GAGTCGACAGCAGGAGACCC	63.5	349
27a	gttcgacctgacaaatgagc	57.3	TCCTCACTTAGCTGATCCGC	59.4	345
27b	acctcaccagtcaacagagc	59.4	CTGCTGGCTTTTCCCCATCC	61.4	339
28	aagagtcagaaactcagccc	57.3	ACATGGAGCTCCGAGGAGGG	63.5	306
30	aagcgccaatgccttgatcc	59.4	ATGCTTTTCCCCTCTCCGGG	61.4	342
32	atcttcccaacggcagcccc	63.5	TCAGCAGCACTGGGACCTGG	63.5	293
33a	ccaagatgacctccttcacc	59.4	AAGGACAGGAGGCTTTACCG	59.4	351
33b	catggccagcttccacagcc	63.5	ACCAGCAAGTCACCGCAGGC	63.5	328
34	actaccgtggagctcaagcc	61.4	ACAACATCTGCAATGTGCCG	57.3	349
35	tattatcgggggacccatgg	59.4	TGGTGAGCTTCACCACATCG	59.4	346
36	taccaggcaatggctggagg	61.4	TTAACAGCAGCCTAGGCCGG	61.4	364
37	atgacatcaccaaccagtcc	57.3	GCTGGAGCGCTTCTTCTGGG	63.5	326
38	acgctccctaatggtaagcc	59.4	AGCAGCTGACAACCTTGGGC	61.4	280
39a	taccgcaactcagttggagg	59.4	CAGAAGCACTCTTTTCTGGG	57.3	391
39b	tcactcgcgcctactacagg	61.4	ACAAATCCACTCTTCACCCC	57.3	361
40b	aggtgcccactgaacaggcc	63.5	GGATGGTCCTGACTTTCCGG	61.4	378
42	tgaacacatccaagcctggc	59.4	GGCATGTGCCTGAGTCCTGC	63.5	338
43	acatcagcaagaggagcacc	59.4	CCGCAATGTCCTTGCTGTCC	61.4	321
44	tgcttcgcaaggcagagggg	63.5	CCAATGAGGCTTTCAGGCGG	61.4	312
