# Supplementary material for: Thousands of Rab GTPases for the Cell Biologist
Source: PLoS Comput Biol. 2011 Oct 13;7(10):e1002217. doi: 10.1371/journal.pcbi.1002217 (PMC3192815; doi:10.1371/journal.pcbi.1002217)
Supplement: Text S1 — Describes how the Rabifier computes Rab family and subfamily scores. (PDF) [file pcbi.1002217.s012.pdf]

**Text S1.** This document describes the computation of the statistical scores generated with each call in phase one and two of the Rabifier (see **Figure 1**).

Generally, the procedure is based on the naive Bayesian classifier {Tom Mitchell, 1997, Machine Learning, Generative and Discriminative Classifiers: Naive Bayes and Logistic Regression}. This well studied probabilistic machine learning approach is one of the simplest yet most performant classifiers in a supervised setting {Naive Bayes classifier}.

The basis for our score is a feature vector corresponding to a given input. In our case, the input is a sequence to be classified, and the features are going to be the output of certain tools (see workflow in **Figure 1**) we feed with the input sequence. In the following, we describe the two distinct steps necessary for the computation: as a prerequisite, we first establish distributions from our reference data or training set, and second, we evaluate them and combine the results to produce a single value per input sequence which represents the actual confidence score. The procedure is equivalent for both Rab family and Rab subfamily scores, with the difference that the Rab family score is binary and only generates two values for the classes Rab and non-Rab, whereas the subfamily score produces one per subfamily in our reference set. For the sake of simplicity, we describe the procedure for the binary case, however, as mentioned all descriptions equivalently apply to the subfamily score.

*Step 1.* The purpose of this phase is to establish how likely certain feature values are under the assumption that the input is a Rab and that it is not. The tools we use to obtain features or measure properties of the input are BLAST {Altschul et al., 1990, J Mol Biol, 215, 403-10} and MAST {Bailey and Gribskov, 1998, Bioinformatics, 14, 48-54} to get the sequence identity, similarity and e-value of the alignment to the best hit in our reference set, and the number and alignment e-value of the RabF motifs {Pereira-Leal and Seabra, 2000, J Mol Biol, 301, 1077-87} respectively. We used the same manually compiled reference set of Rabs and sequences which

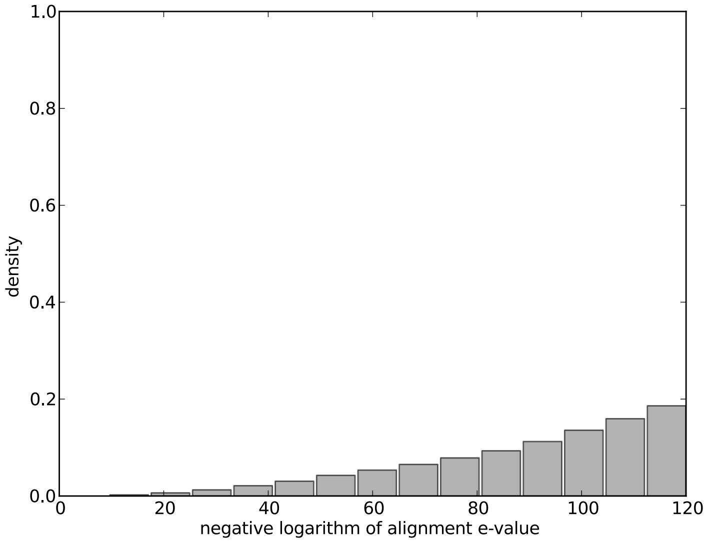


*Figure T1.* Cumulative distribution of the negative logarithm of the BLAST alignment e-value of our reference set of Rabs against itself (self hits excluded).

are not Rabs to measure the values described above, however, to ensure we did not bias the distributions by aligning sequences against themselves we excluded this case for each sequence. The result are two times (both for Rabs and non-Rabs) five histograms (sequence identity, similarity and e-value of the alignment to the best hit in our reference set plus number and alignment e-value of the RabF motifs). For illustration purposes Figure T1 and T2 show two such histograms. Note that unlike for a classical naive Bayesian classifier, we did not fit any distribution to obtain true densities, but used the empirical distributions as they are shown above.

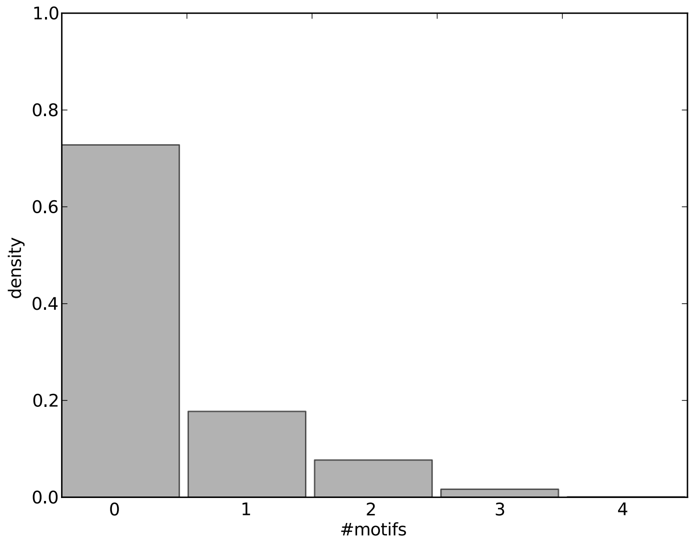


*Figure T2.* Cumulative distribution of number of RabF motifs detected by MAST in the reference set of non-Rabs.

*Step 2.* Given these ten histograms, five per possible outcome (Rab or non-Rab), the computation of the confidence score given an input sequence is straightforward. Once the sequence in question has been BLASTed against Rabifier's internal reference set and MAST has detected the motifs and their e-value, the obtained values are evaluated under both possible outcomes with help of the density functions defined by the histograms. The final score is then obtained by applying Bayes formula:

$$P(C|\vec{F}) = \frac{P(\vec{F}|C)}{P(\vec{F}|C = \text{Rab}) \times P(\vec{F}|C = \text{non-Rab})}$$

where F is the feature vector with five individual components being the sequence identity, similarity and e-value, as well as the motif count and e-value, and C are the possible outcomes or classes, *i.e.* Rab or non-Rab.

Figure T3 shows the distribution of scores we obtained from the application of the Rabifier to 247 genomes taken from the Superfamily database as described in the main manuscript. Note that in a true classification setting, any score below 0.5 would lead to consider a sequence as not being a Rab and vice versa. However, as presented in **Figure 1** and unlike in the second phase, the family score does directly influence the decision of calling a sequence a Rab or not, and is to be understood as a pure confidence level. In fact, the Rabs with scores lower than 0.5 are mostly

accounted for by exceptions as for example very short sequences or those with long strips of masked residues, where motif detection and alignments in general tend to fail.

Figure T4 summarises the result of phase 2 of the Rabifier.

*Figure T3.* Rab family scores obtained for all G-protein family domain containing proteins from the 247 genomes described in the main text. Black bars capture sequences the Rabifier classified as Rabs and are browsable at our public website *www.RabDB.org*, in red are those we classified as not being Rabs. The lower histogram shown the same quantities in log-scale. Sensitivity and specificity refer to the threshold at 0.5 marked by the red dashed line.

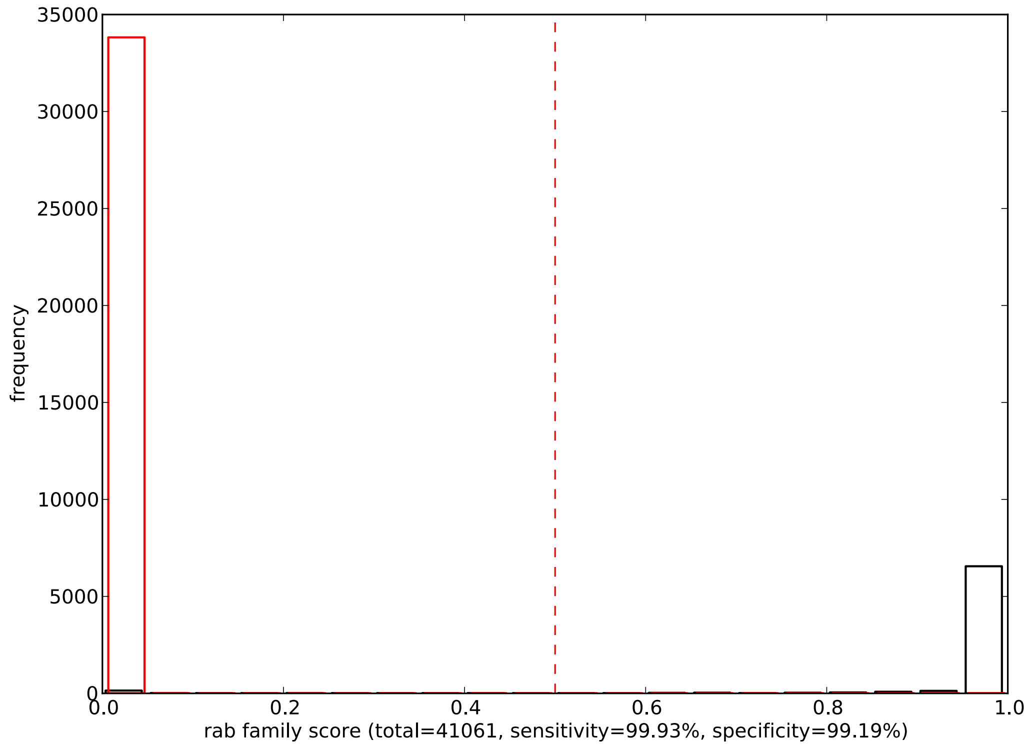
35000
30000
25000
20000
15000
10000
5000
0
frequency
0.0
0.2
0.4
0.6
0.8
1.0
rab family score (total=41061, sensitivity=99.93%, specificity=99.19%)

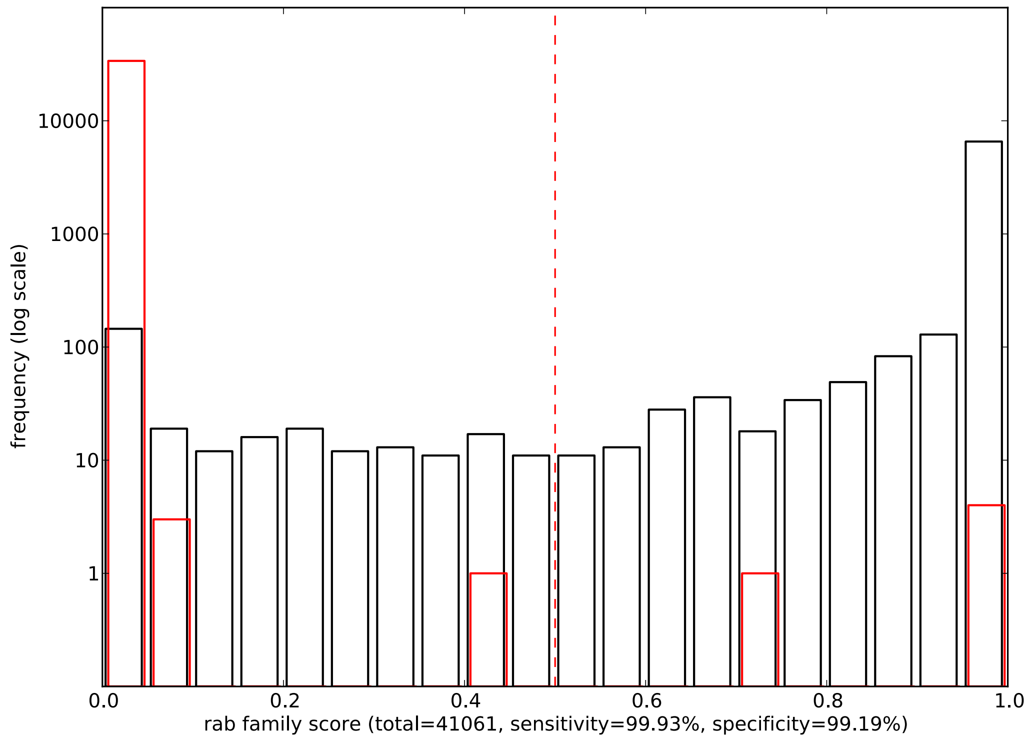
10000
1000
100
10
1
frequency (log scale)
0.0
0.2
0.4
0.6
0.8
1.0
rab family score (total=41061, sensitivity=99.93%, specificity=99.19%)

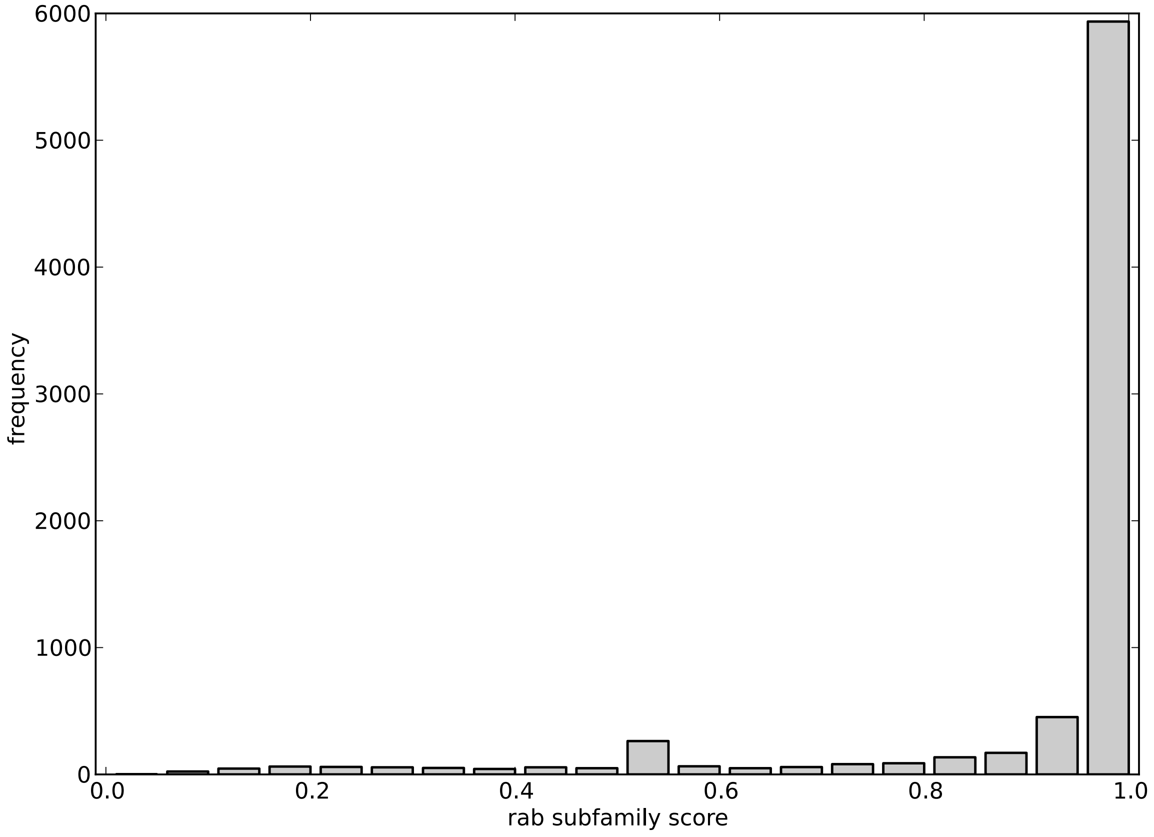


*Figure T4.* Distribution of subfamily scores of the highest scoring subfamily for all Rabs in our database at *www.RabDB.org*.
